# FMCW Radar-Aided Navigation for Unmanned Aircraft Approach and Landing in AAM Scenarios: System Requirements and Processing Pipeline

**DOI:** 10.3390/s25082429

**Published:** 2025-04-11

**Authors:** Paolo Veneruso, Luca Manica, Enrico Miccio, Roberto Opromolla, Carlo Tiana, Giacomo Gentile, Giancarmine Fasano

**Affiliations:** 1 Department of Industrial Engineering, University of Naples Federico II, 80125 Naples, Italy; enrico.miccio@unina.it (E.M.); roberto.opromolla@unina.it (R.O.); g.fasano@unina.it (G.F.); 2 Applied Research & Technology, Collins Aerospace, 00185 Rome, Italy; luca.manica@collins.com (L.M.); giacomo.gentile@collins.com (G.G.); 3 Vision Systems, Collins Aerospace, Wilsonville, OR 97070, USA; carlo.tiana@collins.com

**Keywords:** radar-aided navigation, Advanced Air Mobility, sensor fusion, autonomous approach and landing

## Abstract

This paper focuses on the use of Frequency-Modulated Continuous Wave radars as an aiding source to provide precision navigation during approach and landing operations in Advanced Air Mobility scenarios. Specifically, the radar system requirements are delineated through an analysis of operational constraints defined by regulatory guidelines, including approach trajectories and vertiport infrastructure to ensure compatibility with Urban Air Mobility scenarios. A preliminary radar design is proposed which is integrated within a multi-sensor navigation architecture including a GNSS receiver, an inertial measurement unit, and two cameras. The radar is designed to detect high-reflectivity targets placed in the landing area and uses a matching algorithm to associate these detections with their known positions, enabling reliable corrections to the aircraft navigation state. Radar measurements are tightly integrated into an Extended Kalman Filter alongside data from other sensors, refining the vehicle navigation state estimate and ensuring seamless transitions between long-range and short-range sensing modalities. A high-fidelity simulation environment validates the proposed multi-sensor architecture under different visibility conditions and accordingly disactivating the radar to validate its contribution. The results demonstrate significant improvements in navigation performance when the radar is integrated within the multi-sensor architecture thanks to its important role in providing accurate estimates at high ranges from the landing pattern and during low-visibility operations. The reported statistics of the multi-sensor architecture performance are compared with the assumed required navigation performance in the scenarios of interest, demonstrating the radar contribution and showing the effects of designed radar angular resolution on the multi-sensor architecture.

## 1. Introduction

Approach and landing have consistently been risk-intensive phases in aircraft missions. According to International Civil Aviation Organization (ICAO) statistics [1], approximately 43% of aviation accidents—and up to 50% of fatal incidents—occur during these final stages of flight. Similar trends are evident in helicopter accident reports [2], emphasizing the need to enhance pilot situational awareness to mitigate these risks. Low visibility conditions augment these challenges, necessitating specific sensing architectures for reliable navigation.

This concern is further amplified in the context of Urban Air Mobility (UAM) operations as a part of the wider Advanced Air Mobility (AAM) framework. While well-established solutions for runway landings are widely deployed, their limitations hinder direct integration into vertiport operations. Specifically, the Instrument Landing System (ILS) and the Microwave Landing System (MLS) rely on Radio Frequency (RF) transmitters, which are impractical in UAM scenarios. In fact, urban environments introduce RF interference [3], and the bulky hardware is incompatible with the constrained spaces of vertiports [4]. Additionally, their narrow, rigid beams fail to accommodate the varying approach slopes envisioned for AAM operations [5]. Similarly, the reliability of Satellite-Based and Ground-Based Augmentation Systems (SBAS/GBAS), which generally enhance Global Navigation Satellite System (GNSS) accuracy, decreases significantly in urban scenarios [5,6]. This is mainly due to the poor performance of GNSS receivers in urban environments, where issues such as signal multipath, obstruction, and attenuation degrade the reliability of GNSS signals [7]. Furthermore, jamming and spoofing represent known issues for GNSS-based solutions.

These limitations highlight the need for innovative navigation solutions tailored to UAM approach and landing. Onboard navigation systems, which minimize reliance on ground-based infrastructure, are suited for compact vertiport environments in dense urban areas, where traditional navigation aids fall short. By leveraging simple and inexpensive cues recognizable by onboard sensors, multi-sensor architectures offer a promising solution, combining the strengths of individual sensors to mitigate their respective limitations.

In this context, this paper focuses on defining the system requirements and integrating a Frequency-Modulated Continuous Wave (FMCW) radar within a multi-sensor onboard navigation architecture specifically designed for drones or highly autonomous aircraft without a traditional pilot actively operating the vehicle in AAM scenarios. In these cases, precise navigation is vital to ensure safety and efficiency, especially in complex urban environments. The proposed architecture includes the following:

- A GNSS receiver, which is the main source of aircraft positioning information at long range from the landing area and is integrated with different onboard sensors to avoid the aforementioned limitations that might occur in the final phases of UAM operations at low altitudes;
- An Inertial Measurement Unit (IMU) to propagate the aircraft navigation state, providing high-frequency data and robustness to rapid platform movements;
- Two onboard cameras (namely Forward-Looking, FL, and Down-Looking, DL), which are optimized to provide accurate relative aircraft navigation information in the whole final phase of the approach trajectory;
- An FMCW radar, with the aim to enable vertiport detection at higher ranges (compared to vision-aided navigation) and provide relative navigation information even under reduced visibility conditions.

The following sub-section provides an overview of the state of the art in onboard multi-sensor architectures used to support both manned and unmanned aircraft operations, with a focus on the applications of FMCW radars and the proposed solutions for AAM approach and landing. This is followed by a discussion of the paper’s contributions within the context of the introduced framework.

### 1.1. State of the Art

The main example of certified multi-sensor onboard architecture aimed at supporting manned approach and landing operations is the Enhanced Flight Vision System (EFVS or EVS). As defined by the FAA [8], an EFVS is an aircraft-installed system that provides a visualization of the forward external scene topography, including natural and manmade features. This system can employ imaging sensors, including, but not limited to, Forward-Looking (FL) Infra-Red (IR) cameras, Millimeter Wave (MMW) radiometry, MMW radar, or low-light level image intensification. The EVS utilizes a transparent Head-Up Display (HUD) to overlay flight information, symbology, navigation guidance, and a real-time external scene image for the pilot on a single display, enabling approach and landing operations under low visibility scenarios. These systems are certified by the authorities for aircraft approach and landing on runways, with respective requirements related to runway visibility on the HUD [8,9,10,11]. The sensors adopted in currently certified EVS and installed on many business aviation aircraft include combinations of Short-Wave Infrared (SWIR) cameras, thermal cameras, and visible cameras [12]. Alternative sensors, such as W-band radars, have been explored as potential candidates for EVS, offering improved fog penetration compared to current solutions at the required range for the identification of the needed visual cues [13].

While the primary function of the EVS is to provide visual contact with the approach area to the pilot, the collected data can also be used to extract navigation-relevant information. For instance, autonomous runway detection can be performed using EVS data without relying on synthetic environmental information. Techniques include detecting Light Emission Diode (LED) sources located at the landing area that pulse at half the EVS video rate, enabling the encoding of specific runway identifier codes in the LED patterns, as proposed by Kerr [14]. Alternatively, runway contours can be autonomously extracted from EVS IR images through segmentation and corner detection. Doehler et al. [15], as part of the German Aerospace Research Center (DLR)’s ADVISE-PRO (Advanced Visual System for Situation Awareness Enhancement—Prototype) project, demonstrated the capability to estimate the aircraft’s relative position to the runway by analyzing the extracted geometry without any need for prior runway information. The same project showed that MMW radar images could provide valuable features for runway detection by correlating shadows in radar images with radar data acquired during previous flights in the same area [16]. This work also emphasized the superior weather penetration of MMW radar compared to IR cameras.

Despite its approval for fixed-wing aircraft approaches and landings, the adoption of EVS for civil helicopters presents challenges related to the different motions of these vehicles compared to nominal aircraft. Consequently, a wider Field of View (FOV), not easily adaptable in an HUD, must be covered by the onboard sensors to support the pilots. To address these challenges, the U.S. Army initiated the Degraded Visibility Environment Mitigation (DVE-M) program to improve pilot situational awareness during critical operations under reduced visibility conditions. As part of this program, the DLR implemented an experimental setup featuring an FL LiDAR with a range of 50 m to 1000 m, providing visual cues to pilots via Helmet-Mounted Displays (HMDs) [17]. This work was extended to enhancing obstacle awareness through the combination of sensor data to terrain databases for helicopter off-shore landings [18]. Additionally, the development of symbology and visualization techniques tailored for presenting the synthetic/enhanced data in the HMDs was explored in [19,20]. A combination of LiDAR, an IR camera and a visible camera was proposed by Szoboszlay et al. [21] and tested in the DVE-M program [22] to optimize the visual cues provided to the pilot through an HMD. While exploring alternative sensor suites, the U.S. Army has published the first technical reports concerning the development of a Ka-band Forward-Looking Synthetic Aperture Radar, which aims at supporting helicopter landing areas and obstacle detection through the 3D imaging of the radar data in manned operations [23,24,25].

The advantages of radar, particularly in terms of improved weather penetration, can also be leveraged for autonomous aircraft operations. In this context, an FMCW radar offers notable benefits over traditional pulsed radar systems, making it ideal for Unmanned Aerial Vehicle (UAV) applications. An FMCW radar continuously transmits and receives signals, providing simultaneous high-resolution distance and velocity measurements while operating at lower peak power. This reduces power consumption, an essential factor for UAV platforms with stringent Size, Weight, and Power (SWaP) constraints. Moreover, FMCW radar’s reduced susceptibility to interference enhances its performance in congested urban airspaces.

FMCW radar data can be fused with other onboard sensors to enable navigation functionalities for UAVs. For instance, range and velocity measurements of the 3D scene points detected by an onboard W-band FMCW radar sensor can be fused with IMU data in an Extended Kalman Filter (EKF), using radar-Inertial Odometry (RIO) techniques [26]. Alternatively, Almalioglu et al. [27] proposed integrating an MMW radar operating at the same frequency band with an IMU for indoor navigation, employing a model-free motion dynamics estimation technique in an Unscented Kalman Filter enhanced by Recurrent Neural Networks. For outdoor navigation, RIO (using a 60 GHz radar, V-band) was combined with GNSS position measurements, achieving errors below 3 m over trajectories of up to 300 m in length when flown over urban areas [28]. Other radar fusion methods focus on detecting moving intruders, where K-band radar is paired with stereo cameras to estimate the size, location, and motion of potential threats relative to the host platform [29].

FMCW radars have also demonstrated potential for UAV landing operations. In this respect, an onboard K-band radar was used to detect active beacons and estimate the UAV position relative to the landing point (from Angle of Arrival (AoA) and range measurements), achieving sub-36 cm accuracy within the final 5 m of the approach trajectory [30]. Similarly, the combination of round-trip time-of-flight and Direction of Arrival methods with ground-mounted cooperative antennas enables accurate UAV position estimation during the last 20 m of an approach [31]. Furthermore, a Nadir-Looking (NL) W-band FMCW radar can estimate the UAV position within the final 3 m of a vertical landing trajectory by detecting corner reflectors placed at different heights below a 1 m^2^ acrylic landing pattern [32]. Passive reflectors can also be installed along the edges of a runway to estimate aircraft navigation information [33]. Specifically, the radar data help adjust the approach trajectory of the landing fixed-wing UAV in the horizontal plane based on the relative positions of the reflectors in the captured radar signals.

All these radar-aided autonomous navigation solutions are focused on small UAV or fixed-wing aircraft approach operations without including potential operations to vertiports. However, onboard camera-aided architectures have been explored in the literature to address this challenge for UAM scenarios. One promising approach involves fiducial markers as potential patterns to aid autonomous UAM aircraft landings. Hubner et al. [34] conducted small-scale flight tests to validate the minimum size and type of fiducial marker suitable for vertiport landing pads. Their results demonstrated that 2 m-sided ArUco markers enable reliable detection along the final 60 m of approach trajectories. The same authors proposed a technique to estimate the rotorcraft pose up to 200 m range from the landing pad, leveraging the detection of a yellow circle surrounding the landing area, which encloses the ArUco markers [35]. Alternatively, reference [36] introduced a multi-scale fiducial marker pattern based on customized AprilTags (AT), optimized for vision-based autonomous UAM take-off and landing procedures. Detection is achieved using two high-resolution cameras: one NL and the other oriented 45° downward from a FL configuration.

Recently, NASA has invested significant efforts toward developing well-assessed solutions for accurate vision-aided navigation to vertiports. Kawamura et al. [37] implemented a loosely coupled EKF that combines an IMU with a camera detecting a specific lighting pattern around the landing area. The proposed pattern consists of 110 lights distributed around the Touchdown and Liftoff Area (TLOF), the Final Approach and Takeoff Area (FATO), and around the landing pad. This setup replicates the heliport approach lighting system, covering an area of approximately 350 × 45 m [38]. The method identifies lights in camera frames by reprojecting them into the image plane and matching them to detected corners using either a Harris detector or a Hough circle detector. The iterative COPOSIT (COplanar Pose from Orthography and Scaling with ITerations) algorithm is applied for pose estimation [39]. The effectiveness of this method has been validated through synthetic environments [5] and real flight test data [40]. To support further research, Brown et al. [41] plan to publish a dataset containing real camera and inertial data collected during AAM approach scenarios. This dataset aims to validate the previously introduced EKF and foster the development of innovative computer vision algorithms.

Infrared-based solutions have also been explored to enhance navigation under low-light conditions during UAM approach operations. The authors in [4] implemented an IR-aided navigation solution that uses 16 thermally contrasting point-shaped beacons detected in images acquired by a thermal camera. Structural pattern recognition techniques were applied to identify each beacon, enabling aircraft pose estimation. The study also evaluated the performance of various thermal cameras tested in this configuration. Akagi et al. [42] designed a pattern consisting of 25 blinking point light sources, organized into five legs. This design allows reliable association of the identified points using a camera equipped with an SWIR filter. Results from drone flight tests demonstrated that the method achieves reliable pose estimates within the final 100 m of selected approach trajectories, up to the point (approximately 30 m in these tests) where the entire light pattern is fully framed by the camera. Scholz et al. [35] proposed a dual IR camera setup optimized to continuously monitor the landing area. This setup is planned for testing in upcoming flight campaigns. The same authors [43] introduced an ad hoc image processing technique based on non-linear temperature mapping, which detects landing patterns composed of three 2 m-sided ArUco markers, i.e., the configuration proposed by Hubner et al. [34].

### 1.2. Paper Contributions

Within the introduced sensing architectures, all aimed at providing situational awareness or navigation information to the landing aircraft, a significant gap remains in the development of a well-assessed solution for integrating radars into multi-sensor systems. Radars are recognized for their advantages, including increased range and superior weather penetration compared to alternative exteroceptive sensors. However, their integration into navigation architectures remains an open research topic, mainly due to the potential complexities in interpreting and processing radar data, which is less straightforward than camera data.

In this context, this paper introduces a novel solution to integrate radar systems in multi-sensor navigation architectures, providing robust, weather-resilient navigation information for highly autonomous vehicles and drones in UAM landing scenarios. The resulting onboard navigation architecture is tailored to be integrated with the proposed vertiport design outlined by the European Union Aviation Safety Agency (EASA) [44] for Visual Flight Rule (VFR) operations. This design facilitates seamless integration into the autonomy levels expected for AAM aircraft in forthcoming operations, ultimately enabling fully autonomous approach and landing capabilities [45]. While the vision-aided architecture has been detailed in previous works by the authors [46,47,48], the contributions of this paper specifically tackle the integration of the radar data. The main contributions can be summarized as follows:

- The analysis of airborne radar system requirements in relation to operational constraints, including the approach trajectory and infrastructure defined by EASA regulations for vertiport design;
- The preliminary design of an FMCW radar, compliant with the identified requirements, and the definition of an ad hoc radar signal processing pipeline to extract navigation information from collected data. This includes a strategy to identify and match targets of interest located within the vertiport area;
- The definition of the multi-sensor navigation architecture, including the integration of matched radar target coordinates into the navigation filter architecture in a tightly coupled manner. This integration supports different operational modes based on the sensors contributing to the correction step of the navigation filter;
- The development and adoption of a high-fidelity simulation environment to simulate data collected by onboard exteroceptive sensors and validate the complete navigation architecture. The adoption of a physics-based simulator enables detailed modelling of radar interactions with the environment, incorporating realistic representations of object properties, noise patterns, and artifacts such as multipath reflections and clutter. These simulations realistically assess the contribution of radar data to the proposed navigation architecture, including scenarios with reduced visibility (e.g., intense fog), where camera activation occurs later compared to nominal visibility conditions.

A preliminary version of the radar system requirements and the FMCW radar integration into the multi-sensor architecture were presented in [49,50]. This paper represents a significant advancement compared to previous works owing to the following:

- Analyzing radar system requirements with consideration of their impact on the autonomous AAM vehicle in terms of SWaP characteristics;
- Providing a detailed design of the radar system, ensuring compliance with the minimum identified requirements, followed by its simulation. This process effectively verifies the contribution of critical parameters, such as radar azimuth resolution, which were not analyzed in previous works;
- Incorporating statistical simulations within the high-fidelity simulation environment, enabling an evaluation of the navigation performance across different test cases. This approach assesses the designed radar’s contribution to the multi-sensor architecture relative to the specifically assumed required performance.

The paper is organized as follows: Section 2 provides an analysis of the radar system, addressing operational constraints and defining system requirements while presenting the preliminary design of the FMCW radar used in this study. Section 3 outlines the implemented radar signal processing approach and the subsequent integration of radar data into the multi-sensor navigation pipeline. Section 4 describes the high-fidelity simulation environment used to simulate onboard sensor data, validate the radar contributions to the navigation solution, and identify potential improvements to the navigation architecture and radar design. Finally, Section 5 presents the conclusions and outlines future activities aimed at enhancing this work.

## 2. FMCW Radar System

This section discusses the design of an onboard FMCW radar, starting with an analysis of the operational constraints dictated by current regulations for AAM approach and landing procedures. Specifically, the assumed approach trajectory and the ground infrastructure installed near the landing pad, which enable radar-aided navigation, are presented. These assumptions serve as key constraints for defining the requirements of an effective radar system tailored to AAM scenarios. Finally, a preliminary radar system design is introduced, providing a foundational model for implementing signal processing strategies and validating its contributions to navigation performance.

### 2.1. Operational Constraints in AAM Scenarios

The assumptions regarding the approach trajectory and ground infrastructure in this work are based on guidelines provided by EASA for vertiport design to support VFR UAM operations [44]. While primarily focused on manned operations, these guidelines acknowledge the critical role that exteroceptive sensors will play in early UAM implementations. Notably, such sensors are recognized as potential aids to expand the FOV provided by cockpit windshields, ensuring visual contact with the landing area during the final phase of approach procedures. This need for an expanded FOV arises from the approach trajectory described in the guidelines, particularly for vertiports situated in urban environments. This trajectory is constrained by a safety volume, termed the Obstacle Free Volume (OFV), above which the Vertical Take-Off and Landing (VTOL) aircraft must operate. Based on this, the approach trajectory assumed in this study consists of three main steps designed to comply with the OFV limit.

**Figure 1:**
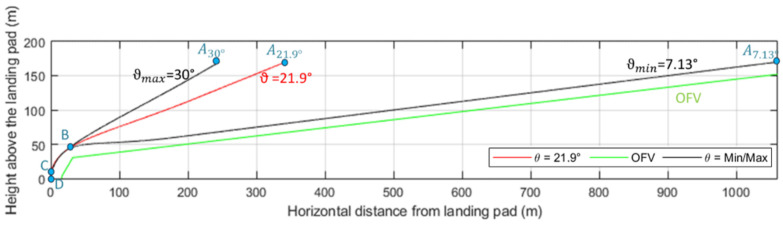
Obstacle Free Volume (OFV) defined by European Union Aviation Safety Agency (EASA) [44] and potential approach trajectories (points A-B-C-D) as a function of the slope angle (ϑ).

- A first descent at constant slope (ϑ) starting from the cruise altitude. The EASA regulations extend the OFV up to 152 m above the vertiport; thus, this work assumes a cruise altitude ($h_{CRUISE})$ of 170 m to account for potential vertical positioning errors of the VTOL aircraft. The slope value is determined by the aerodynamic and power constraints of the landing vehicle, ranging from a minimum of 7.13° (per EASA guidelines) to a maximum of 30° [51]. For this study, an intermediate value of slope among these limits (i.e., *ϑ =* 21.9°) is assumed. This trajectory phase is defined by points A-B in Figure 1, illustrating the OFV and trajectory variations as a function of slope.
- A second and steeper descent aligning the vehicle horizontally with the center of the landing pad. The endpoint of this segment (Point C in Figure 1) is located 3 m above the landing area, horizontally centered over the pad.
- A vertical descent for the final 3 m of approach. This phase concludes with the aircraft reaching Point D, located at the center of the landing pad (i.e., the ideal touchdown point).

The simulated approach trajectory assumes velocities that gradually decrease from the cruise speed (consistent with typical VTOL vehicle prototypes like the Volocopter Volocity [52]). During the final two phases, vertical velocity is limited to mitigate Vortex Ring State (VRS) effects. The total duration of the simulated approach trajectory depends on the slope of the first phase, with a duration of 29.5 s for the selected slope.

**Figure 2:**
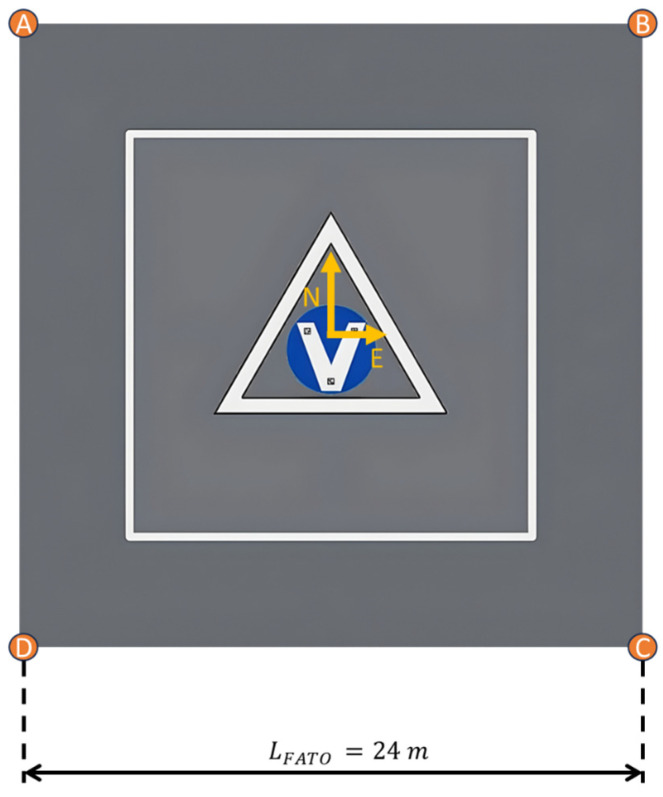
Selected landing pattern with the 4 high Radar Cross Section (RCS) targets placed at the corners of the Final Approach and Take-Off (FATO) area, in orange (A, B, C, D). The North-East axes of the local reference frame are reported in yellow, the Down axis points toward the pattern.

The design of the landing area imposes additional operational constraints for the radar system. Specifically, the area must be detectable in radar data. The adopted solution involves the installation of high Radar Cross Section (RCS) targets on the ground, providing robust radar returns that can be matched with their pre-determined positions. EASA defines a FATO area as the area surrounding the touchdown point, which must remain obstacle-free. The size of this square area depends on the maximum dimensions of the operating vehicles and is assumed to be $L_{FATO}=24m$ for this work. The high RCS targets are positioned at the four corners of the FATO area, as illustrated in Figure 2. These targets’ positions within the local reference frame are a priori known by the onboard navigation system. Additionally, the multi-scale landing pattern, delineated by the white square in Figure 2, incorporates visual key points, which can be detected in image frames to provide vision-based navigation measurements.

Two primary reference frames are utilized in this work.

- A North-East-Down (NED) reference frame centered in the landing pattern, which serves as the local reference frame. The VTOL aircraft trajectory, as well as the coordinates of the radar targets and visual key points, are defined relative to this reference frame;
- A Radar Reference Frame (RRF) with its origin located in correspondence of the onboard FMCW radar. The RRF has its x–y plane aligned with the phased radar antenna elements, while the *z*-axis is the radar boresight direction. The position of specific targets in this reference frame can also be expressed in polar coordinates, defined by range, azimuth (Az), and elevation (El). Azimuth is the horizontal angle between the radar boresight (*z*-axis) and the target, measured in the x–z plane, positive in clockwise direction where Az = 0° means the target is directly along the boresight. Elevation is the vertical angle between the *z*-axis and the target, measured in the y–z plane, with positive angles above the x–z plane and negative angles below it. The antenna elements of the designed phased array and the collected radar data are expressed relative to this reference frame.

### 2.2. Minimum Radar System Requirements

#### 2.2.1. Radar Resolution Requirements

A radar system determines the position of a target in the three RRF coordinates: range (distance) and two angular measurements, elevation and azimuth. For effective operation during landing, the radar is expected to clearly distinguish individual targets of interest throughout the approach phase in which the system is operative. This requires that the targets are separable in at least one measurement dimension, such as range, elevation, or azimuth.

**Figure 3:**
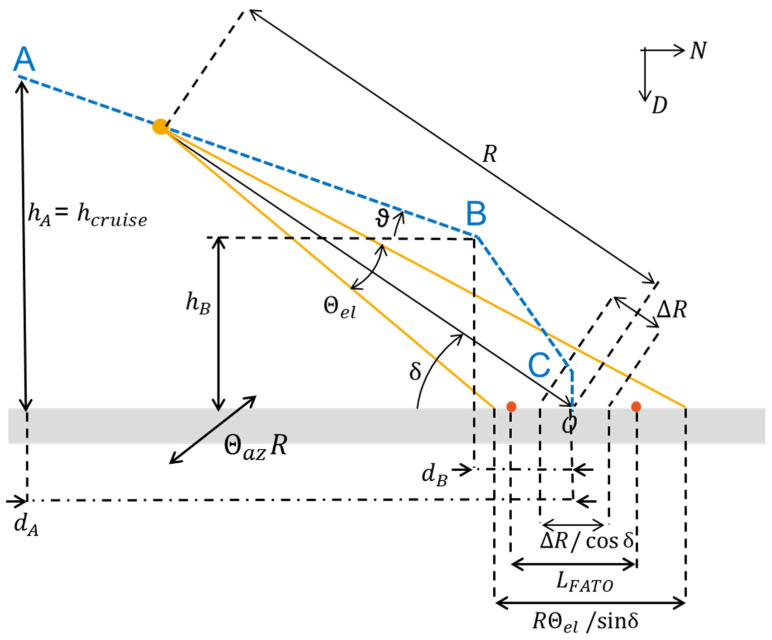
Approach trajectory projected on the North-Down plane, and radar beam extended geometry (delimited by the yellow lines along the elevation direction). The blue dotted line reproduces the aircraft approach trajectory, the orange circles are the high RCS targets. Figure is not to scale.

The radar’s ability to distinguish targets based on range is defined by its range resolution, ΔR, expressed as [53]:

(1) $$\Delta R=\frac{c_{0}}{2B}$$

where $c_{0}=3\times 10^{8}$ m/s represents the speed of light in a vacuum and, B is the radar signal bandwidth. The radar’s capacity to distinguish targets in angular terms, known as cross-range separation, is influenced by the area illuminated by the main beam, $A_{s}$. The computation of $A_{s}$ depends on the grazing angle (δ), which is the angle between the radar line-of-sight and the terrain (see Figure 3). In the following analysis, the grazing angle is assumed to be dynamically adjusted to ensure the radar beam remains directed toward the center of the landing pattern at each point. This corresponds to a scenario where the radar is mounted on a gimbal system that modifies the orientation of the transmitted beam according to the aircraft’s position along the trajectory. As a consequence, the grazing angle influences how the radar beam interacts with the ground, affecting key performance parameters such as the illuminated area $A_{s}$, resolution capabilities, and target separation. Higher grazing angles typically lead to a more focused footprint of the radar beam on the ground, improving angular resolution, while lower grazing angles may increase the extent of the illuminated area, potentially affecting detection performance.

In the case of high grazing angles, $A_{s}$ is calculated as [53]:

(2) $$A_{s}=\frac{R^{2}\Theta_{az}\Theta_{el}}{\sin\delta }$$

where R is the distance between the radar and the terrain surface, and $\Theta_{az}$ and $\Theta_{el}$ are the azimuth and elevation 3 dB beamwidths of the radar antenna, respectively. Conversely, in the case of low grazing angles, $A_{s}$ becomes [53]:

(3) $$A_{s}=\frac{R\Theta_{el}\Delta R}{\cos\delta \;}$$

At the start of the approach phase, the grazing angle is typically low, making Equation (3) applicable. Under these conditions, the required range resolution and azimuth beamwidth are constrained by the following conditions:

(4) $$\begin{array}{c} \Delta R\leq L_{FATO}\cdot \cos\delta \\ \Theta_{az}\leq \frac{L_{FATO}}{R}\;\;\;\;\;\;\;\;\; \end{array}$$

where $L_{FATO}$ represents the spacing between the reference targets along the North and East axes. As the first phase of the approach ends (i.e., at point B of Figure 1), the grazing angle reaches its maximum value, $\delta_{max}$. Consequently, the range resolution must satisfy the condition:

(5) $$\Delta R\leq L_{FATO}\cdot \cos\left(\delta_{max}\right)=11.26\;m$$

In this scenario, $L_{FATO}=24\;m$, while $\delta_{max}=\mathrm{atan}\left(h_{B}/d_{B}\right)=62.30^{{}^{\circ}}$ where $h_{B}=40$ m and $d_{B}=21\;m$. Using Equation (1), the minimum radar signal bandwidth required is $B_{C}\geq 13.40\;MHz$.

To calculate the azimuth beamwidth, the largest value of $R=\sqrt{h_{CRUISE}^{2}+d_{A}^{2}}=400\;m$, observed at the start of the approach procedure, is considered:

(6) $$\Theta_{az}\leq \frac{L_{FATO}}{R}=3.44^{{}^{\circ}}$$

If it is acceptable to distinguish the targets along the azimuth direction only in a second phase of the approach trajectory, the requirement on the corresponding beamwidth becomes less strict (e.g., for R=300 m the required value is $\Theta_{az}\leq 4.6^{o}$). This relaxed condition may also be applicable to navigation architectures that require input from only one matched target rather than identifying all targets within the radar acquisition. The implemented navigation system in this study relies on such an assumption.

Finally, the elevation beamwidth is derived assuming that the autonomous VTOL aircraft approaches with a low grazing angle (i.e., in point A computed with 21.9° of first descent slope). Using Equation (3), it is given by:

(7) $$\Theta_{el}\leq \frac{L_{FATO}}{\Delta R\cdot R}\cdot \cos\delta$$

Calculating $\delta =\mathrm{atan}\left(\frac{h_{CRUISE}}{d_{A}}\right)=26.3{}^{\circ}$, while considering R=400 m and selecting ΔR = 3 m, which is a feasible value for navigation purposes, the elevation beamwidth becomes $\Theta_{el}\leq 15.1^{{}^{\circ}}$.

The calculated values represent the minimum requirements necessary for distinguishing reference targets during the landing approach. However, stricter specifications may be needed to ensure compatibility with the required performance of the resulting navigation system.

#### 2.2.2. Radar Frequency Band Analysis

The choice of radar frequency bands is influenced by the need for compact onboard installation, high antenna gain, and narrow beamwidth. This study limits the analysis to frequencies above 10 GHz. Table 1 summarizes the radar frequency bands that are most suitable for addressing the design requirements, i.e., the Ku, K, Ka, and W-bands. The Ku-band, currently designated for Aeronautical Radio Navigation Service (ARNS), supports radar systems for airborne radionavigation [54]. Meanwhile, the Federal Communications Commission (FCC) has allocated the K- and Ka-bands for radionavigation purposes [55]. Although the W-band has not yet been allocated for radionavigation, its potential use in airborne EVS has been explored in recent studies [13].

The evaluation of radar frequency bands accounts for various physical effects that reduce signal strength, including free-space path loss, atmospheric attenuation, and rain-induced signal degradation. Subsequently, the influence of the radar frequency band on the SWaP characteristics of the radar system is analyzed, evaluating the size of the antenna and the minimum required power.

Free-space path loss, denoted as $\gamma_{FSPL}$, quantifies the reduction in power of an electromagnetic (EM) signal as it travels through free space. It is expressed as the ratio of the received power $P_{RX}$ to the transmitted power $P_{TX}$ for two isotropic antennas separated by a distance r [56]:

(8) $$\gamma_{FSPL}=\frac{P_{RX}}{P_{TX}}={\left(\frac{\lambda }{4\pi r}\right)}^{2}={\left(\frac{c_{0}}{4\pi fr}\right)}^{2}$$

For convenience, this formulation expresses FSPL in its non-logarithmic form. Here, $\lambda =c_{0}/f$ represents the signal wavelength, where f denotes the signal frequency. For narrowband signals, the reference is typically the center frequency $f_{c}$. Table 2 summarizes the free-space path loss values for various cases. Lower frequencies experience reduced losses, with differences of up to 17 dB between the Ku and W-bands, while the K and Ka-bands exhibit comparable losses. Regarding distance, power losses increase by 6 dB/oct. It is important to highlight that $\gamma_{FSPL}$ signifies a one-way loss, meaning that radar signals will experience a 12 dB/oct decay when considering the round trip between the radar and the target. Consequently, lower frequencies are advantageous due to their reduced free-space power losses.

At frequencies exceeding 10 GHz, RF signals can experience significant attenuation due to absorption of resonance lines associated with oxygen, water vapor, and nitrogen gas. This cumulative effect is represented by the atmospheric loss coefficient $\gamma_{atm}$, which is calculated as the sum of two components, i.e., $\gamma_{0}$ and $\gamma_{w}$ [57]:

(9) $$\gamma_{atm}\left(f,T,p,\nu \right)=\gamma_{0}\left(f,T,p\right)+\gamma_{w}\left(f,T,p,\nu \right)$$

In Equation (9), f represents the frequency of radar signal, p  the air pressure, T the air temperature and ν the water vapor density. $\gamma_{0}$ and $\gamma_{w}$ denote the specific attenuation caused by dry air (including oxygen, pressure-induced nitrogen, and non-resonant Debye attenuation) and water vapor, respectively. Further details on the calculation of these coefficients are provided in [57]. Table 3 presents the values of $\gamma_{atm}$ for the frequencies of interest, assuming p=1013.25 hPa, T=288 K and $\nu =7.5\;gr/m^{3}$. Notably, the loss coefficient in the Ka-band is lower than in the K-band, as the Ka-band lies within an atmospheric window centered around 35 GHz. Nevertheless, for the detection ranges under consideration (below 500 m), the resulting losses are negligible across all candidate radar frequency bands.

Finally, rain weakens the radar signal through a coefficient $\gamma_{r}$ (measured in dB/km), which is calculated as [58]:

(10) $$\gamma_{r}=\beta \left(f\right)r_{f}^{\alpha \left(f\right)}$$

In this context, $r_{f}$ denotes the rain rate (in mm/h), while $\alpha \left(f\right)$ and $\beta \left(f\right)$ are coefficients obtained through curve-fitting based on power-law approximations from scattering calculations. Table 4 summarizes the values of $\gamma_{r}$ for various frequency bands and selected rain rates, ranging from light to moderate rainfall. The rain loss coefficient increases with both the frequency and the rain rate. Considering the short detection range required for landing and approach operations (i.e., R ≤500 m), rain losses are acceptable in the Ku-, K-, and Ka-bands. However, the W-band might not satisfy the “all-weather conditions” requirement for the higher ranges to the vertiport.

#### 2.2.3. Radar Antenna

Radars are equipped with antenna systems designed to achieve high gain and narrow beamwidth, typically employing either reflector antennas or electronically scanned antenna arrays. For airborne radar applications, electronically scanned arrays are preferred due to their low-profile design, which aligns with aerodynamic constraints, and their ability to scan electronically rather than mechanically.

For a planar array situated in the xy-plane of the RRF with $N_{x}\times N_{y}$ uniformly excited elements and inter-element spacings $d_{x}$ and $d_{y}$ along the x- and y-axis, respectively, the beamwidth (in radians) of the main beam directed broadside in the azimuth plane ($\Theta_{az})$ and elevation plane $\Theta_{el}$ is given by [59]:

(11) $$\Theta_{az}\approx \frac{0.886\lambda }{N_{x}\cdot d_{x}};\;\Theta_{el}\approx \frac{0.886\lambda }{N_{y}\cdot d_{y}}$$

where λ  is the operating wavelength of the array. Setting $d_{x}=d_{y}=\lambda /2$ and using the values estimated previously of maximum angular beamwidths ($\Theta_{az}=3.44{}^{\circ}$, $\Theta_{el}=15.1{}^{\circ}\;at\;R\;=\;400\;m)$, the minimum number of elements $N_{x}$ and $N_{y}$ turns out to be: $N_{x}=29.5$ and $N_{y}=6.72$. Additionally, for manufacturing requirements the number of the antenna array elements is typically a multiple of four, so $N_{x}=32$ and $N_{y}=8$. An alternative to relax the $N_{x}$ requirement and reduce the number of antennas is to allow less stringent azimuth discrimination of targets during initial acquisitions along the approach trajectory. As previously estimated, at R = 300 m, the corresponding maximum azimuth beamwidth is $\Theta_{az}\leq 4.6^{{}^{\circ}}$ leading to a reduced minimum number of antennas $N_{x}=22.07$ (rounded up to 24). This limited number of antennas reduces the impact of the radar system on the unmanned/autonomous VTOL aircraft in terms of SWaP characteristics.

The antenna gain G and the beamwidths in the azimuth $\left(\Theta_{az}\right)$ and elevation plane ($\Theta_{el})\;$(both measured in degrees) are related by [60]:

(12) $$G=\frac{26400}{\Theta_{az}\Theta_{el}}$$

Substituting the beamwidth values for $N_{x}=24$ and $N_{y}=8$ into Equation (12), the gain is calculated as G=26.91 dBi.

In airborne radar systems, antenna size is a critical factor due to strict space constraints. Higher radar frequencies are preferred because the aperture size of a planar array scales inversely with $1/\lambda^{2}$. For example, Table 5 illustrates the dimensions of an 24×8 rectangular planar antenna array with inter-element spacing of λ/2 across the four considered radar frequencies. Antenna sizes range from tens of centimeters in the Ku-band to a few square centimeters in the W-band (which could be the ideal choice for small drones). As a result, K-, Ka-, and W-band radars are well-suited for integration into autonomous VTOL aircraft, while the Ku-band may be too bulky for some platforms.

#### 2.2.4. Transmitted Power Computation

The minimum transmitted power $\;P_{TX}^{min}$, necessary for detecting reference targets at the maximum distance $R_{max,\;Rad}$, can be determined by rearranging the radar equation in its basic form:

(13) $$P_{TX}^{min}=\frac{SNR_{min}{\left(4\pi \right)}^{3}R_{max,Rad}^{4}Lk_{B}T_{o}B_{c}F}{G_{TX}G_{RX}\lambda^{2}\sigma_{t}}$$

In Equation (13), $SNR_{min}$ represents the minimum required Signal-to-Noise Ratio (SNR). The term L accounts for various radar losses, including beam shape, conversion, and antenna losses. $k_{B}$ is the Boltzmann constant, $T_{0}$ is the system temperature, B represents the band of the radar signal prior to signal processing. The receiver noise figure is F, while $G_{TX}$ and $G_{RX}$ are the gains of the transmitter and receiver antennas, respectively. The radar signal wavelength is λ, and $\sigma_{t}$ is the RCS of the target, which varies based on parameters such as the target size, shape, signal frequency, polarization, and angle of incidence.

For simplicity in initial analysis, only the RCS dependency on the frequency is considered. Specifically, a reference RCS of a potential target, $\sigma_{t,ref}$ is defined for the Ku-band, and the RCS at other frequencies is scaled using:

(14) $$\sigma_{t}\left(f\right)=\sigma_{t,ref}\cdot \frac{f}{f_{ref}}$$

where f represents the frequency, and $f_{ref}=13.325\;GHz$ corresponds to the Ku-band frequency. It is important to highlight that Equation (14) incorporates an RCS dependency of 1/λ.

To provide a rough estimate of the required power, Table 6 summarizes the values calculated using Equation (13) with the following parameters: $R_{max,Rad}=400\;m$, $SNR_{min}=13\;dB$, L=10 dB, $T_{0}=290\;K$, B=20 MHz, F=10 dB, $\sigma_{t,ref}=6\;dBsm$ and $G_{TX}=G_{RX}=26.91\;dBi.$ The minimum required power ranges from 0.376 W for the Ku-band up to 2.64 W for a W-band radar.

### 2.3. Preliminary Radar System Design

**Figure 4:**
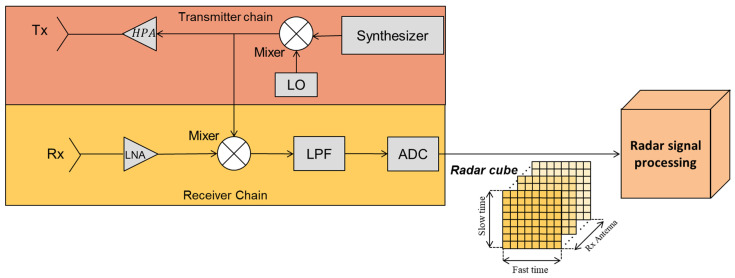
Frequency-Modulated Continuous Wave (FMCW) radar transmitter and receiver chain—simplified flowchart. The synthesizer, the High-Power Amplifier (HPA), and the Local Oscillator (LO) are the main blocks of the transmitter chain. The Low Noise Amplifier (LNA), the Low Pass Filter (LPF), and the Analog to Digital Converter (ADC) are the main components of the receiver chain. A 3D radar cube is the final output combining the data from the 192 ADCs of the 24 × 8 receiver chains. This cube serves as the input for the Radar Signal Processing block.

A generic FMCW radar, designed to meet the previously computed minimum requirements, is employed to detect the range, angular position, and velocity of targets (refer to Figure 4). This radar emits periodic pulses with a frequency that changes linearly over time. The range and radial velocity of the targets are calculated by measuring the frequency shift between the transmitted and received signals, while beamforming techniques are employed to determine the angular position of the targets in both elevation and azimuth.

**Figure 5:**
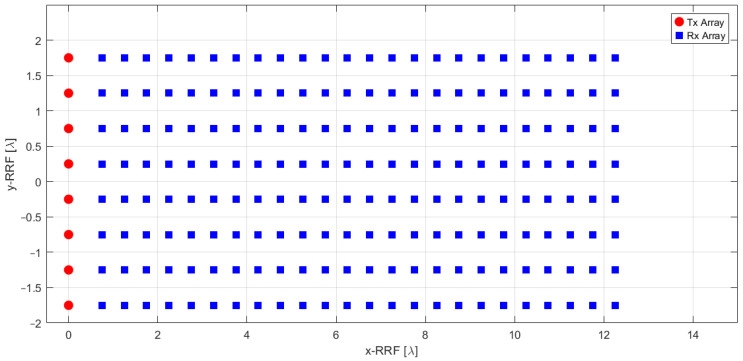
Antenna system geometry in Radar Reference Frame (RRF) (in the plane at z = 0). The spacing between the Tx and Rx arrays (0.75 × λ) is chosen for representational purposes and does not influence the performance of the transmission and reception chains.

The radar antenna system depicted in Figure 5 is assumed to be mounted at the front of the unmanned/autonomous aircraft structure. The transmitter chain utilizes an active electronically scanned linear array consisting of $N_{TX}=8$ elements, which are evenly spaced along the y-axis with an inter-element distance of d=λ/2. Here, $\lambda =c_{0}/f_{c}$, where $f_{c}$ is the center frequency of the radar waveform. According to the previously defined radar system requirements, the Ka-band ($f_{c}=32.85\;GHz$) is selected for this analysis, due to its balance between low atmospheric absorption and required antenna sizing. On the receiver side, a planar array comprising $N_{RX}=24\times 8$ elements, uniformly spaced at d=λ/2, is employed. Each radiating element exhibits a patch-like radiation pattern, achieving a beamwidth of approximately 75° in both elevation and azimuth.

The transmitter array geometry enables beam scanning exclusively in elevation. The radiated antenna array pattern exhibits a beamwidth of roughly $\Theta_{el}$ = 12.5° in elevation and $\Theta_{az}$ = 75°, in azimuth. The radar FOV in elevation ($FOV_{el}$) spans $\pm 36^{{}^{\circ}}$, enabling it to detect the targets of interest located on the ground along the approach trajectory together with aerial objects such as drones or other aircraft flying in the vertiport area. It is important to highlight that even if the radar system design enables the identification of the moving targets in radar data, this task is beyond the scope of this work. The receiver performs beamforming during the signal processing phase, allowing for coherent data combination across multiple antennas (also referred to as phase centers [61]). This process enhances angular selectivity by analyzing the echoes’ AoA.

The FMCW radar emits a sawtooth waveform as its reference signal. The waveform parameters—chirp time ($T_{c}$), the repetition time ($T_{R}$), the frame time ($T_{f}$), the minimum and maximum chirp frequencies ($f_{min}$ and $f_{max}$), and the power of the single chirp $(P_{c}$)—are configured to achieve detection of targets up to a range of $R_{max,Rad}=500\;m$. The system is designed to identify targets moving at speeds up to $v_{max}=200\;Km/h$ while providing a range resolution of ΔR=3 m and a speed resolution of Δv=4 Km/h.

## 3. Radar Data Processing and Fusion Architecture

**Figure 6:**
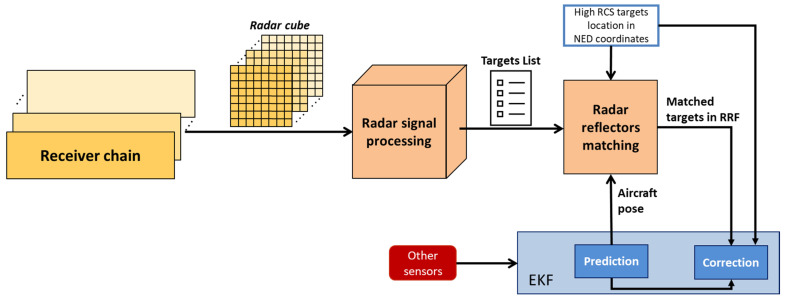
Flowchart of the implemented processing to integrate radar signal in the implemented Extended Kalman Filter (EKF).

This section details the radar signal processing pipeline and the subsequent integration of the radar data into the navigation architecture. As anticipated in Figure 4, the output data obtained from the receiver chain is represented by a 3D radar cube in terms of Fast Time, Slow Time and receiver antenna channels. This tridimensional data is acquired for each beam scan along the elevation angle and is processed by the *radar signal processing* block, which reduces the data to a matrix list of potential targets. The *radar reflectors matching* block then identifies radar targets of interest associating a set of known NED coordinates to each of them. These matched detections are tightly integrated in the multi-sensor EKF, providing corrections to the autonomous VTOL aircraft navigation state. Figure 6 summarizes the main steps involved in integrating FMCW radar data into the navigation architecture.

### 3.1. Radar Signal Processing and Targets Detection

**Figure 7:**
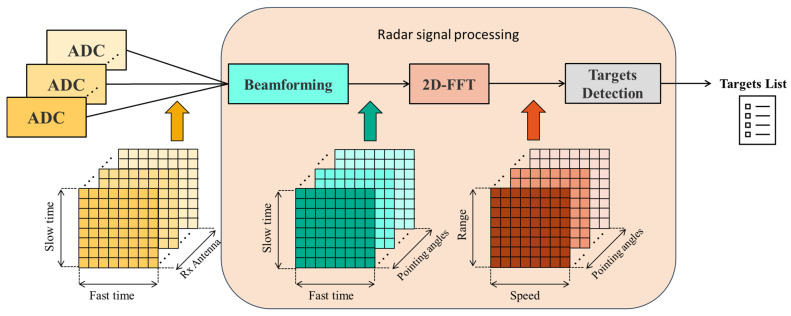
Radar signal processing block—simplified steps to obtain the targets list.

Once echoes from the targets are received by the antennas, they are processed through the receiver chain, conditioned, and finally sampled by the ADC. The resulting data are analyzed to estimate the range, velocity, and angular position of the targets. Figure 7 illustrates the simplified radar signal processing workflow used in this work. The ADC samples are organized into a radar data cube, structured with chirp samples on the rows, chirp numbers on the columns, and receiver channels representing the antennas along the third dimension.

Beamforming is then applied to the received signals from multiple antennas, combining them to emphasize signals from specific angular positions and producing the effect of a directionally focused beam. The following 2D Fast Fourier Transform (FFT) processes the beamformed data to separate target information in terms of range and velocity, producing a range-Doppler map. The speed-range maps are exploited to determine the range and speed as well as the angular position of the targets. The former information (speed and range) is determined by means of a peak detection algorithm coupled with a Cell-Averaging Constant False Alarm Rate (CFAR) algorithm [61], while the angular position is computed considering the matrix in which the identified target shows the maximum amplitude (the one with stronger radar echoes). Consequently, for each elevation step, this process generates a list of potential target returns, which includes:

- RRF polar coordinates (Az, El, Range);
- Radial velocity;
- Signal peak power;
- Background noise level.

Every 0.1 s, the radar completes a full azimuth scan at the current elevation angle, producing a new list of detected targets. The scan time was determined to be consistent with the frame time ($T_{f}$) needed to fulfill the radar performance with respect to maximum detectable range, maximum detectable speed and speed resolution, previously reported in Section 2.3. Moreover, the selected value (0.1 s) aligns with the typical performance of radar systems used in automotive applications and satisfies the update rate requirements for the considered application. The list of targets is passed to the *radar reflector matching* block. Each list includes all the accepted returns detected during the azimuth scan, including clutter and signals from objects in the radar FOV that are not relevant to the navigation architecture.

### 3.2. Radar Reflector Matching

**Figure 8:**
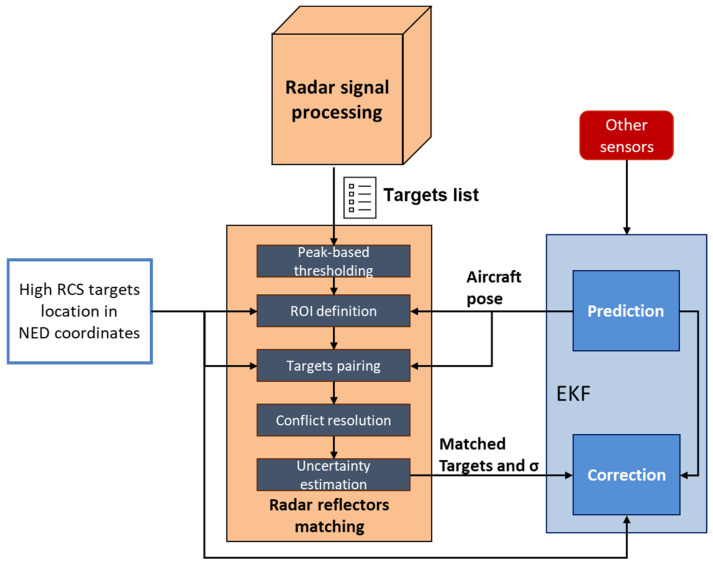
Flowchart for radar reflectors matching in radar data.

The *radar reflector matching* block processes the list of potential targets to extract only those corresponding to the high RCS reflectors placed at the corners of the FATO area shown in Figure 2. The output of the radar reflectors matching algorithm consists of target detections in the RRF (Az, El, Range), paired with corresponding high RCS targets of interest and the associated uncertainties. The main steps of the algorithm are outlined in Figure 8 and described below:

1. Peak-based thresholding. A threshold based on the targets’ signal peak power filters out target returns with lower intensities than the ones expected for the targets of interest. The threshold can be selected through a calibration process by observing target returns at the relevant ranges. This threshold is here set to a value marginally below (e.g., −1 dB) the expected return power of the farthest detected target but may adapt with range as sufficient data becomes available to characterize targets’ returns.
2. Region of Interest (ROI) Definition. The known coordinates of the radar reflectors in the NED reference frame are used to define an ROI that is expected to include the target detections. The ROI is initially defined in NED by projecting the reflector locations and then expanded based on the navigation uncertainty. Specifically, the a priori state covariance is used to determine confidence bounds, such as a 95% interval, by extending the ROI along each axis in North-East-Down coordinates. Once the expanded ROI is established in NED, its vertices are transformed into the RRF. The list of detected radar targets, which is expressed in RRF, is then filtered by retaining only those within the transformed ROI. This ensures that the target selection process accounts for navigation uncertainty, preventing the exclusion of valid detections due to estimation errors. The navigation state covariance, used for ROI expansion, is propagated in the prediction step of the EKF using Van Loan’s method [62] and updated in the correction step through the measurement covariance matrix and Kalman gain [63].
3. Targets Pairing. The remaining targets, including reflections from both the targets of interest and nearby objects/clutter, are matched to the reprojected radar reflector coordinates in the RRF. Each radar return is associated with the closest radar reflector by minimizing the distance to the four reprojected coordinates. The spacing of 24 m between the reflectors (i.e., $L_{FATO}$) ensures reliable matching, even in the presence of navigation state errors.
4. Conflict Resolution. When multiple returns are paired to the same reflector, only the strongest return is retained, based on the assumption that the high RCS targets installed in the landing area generate the highest intensity returns compared to nearby objects.
5. Uncertainty Estimation. Detection uncertainties are calculated using the formulas [61]:

(15) $$\begin{array}{c} \sigma_{R}=\frac{\Delta R}{\sqrt{2\cdot SNR}}\;\;\;\;\;\;\;\;\;\;\;\;\;\;\;\;\;\;\\ \sigma_{A}=\frac{\Theta_{A}}{\sqrt{2\cdot SNR}};A=Az,El \end{array}\;$$

Here, $\Theta_{A}$ is the angular beamwidth, and the SNR is derived as the ratio between signal peak power and background noise. For high SNR values (above 25 dB), the additional error sources, such as fixed random error and bias error (which can both be empirically estimated as 1/25 × Δ*R*, 1/25 × $\Theta_{A}$ are summed to the estimated uncertainties [53].

As previously discussed in the context of the required azimuth beamwidth, the concept of tightly integrating radar detections into the navigation filter enables flexibility in the number of matched radar reflectors identified by the algorithm. Specifically, the proposed architecture works with just one correctly matched target.

### 3.3. Radar-Aided Extended Kalman Filter

The implemented navigation architecture estimates the VTOL aircraft state (i.e., **x**), which comprises the attitude (**Ψ**), the position (**p**^n^), and velocity (**v**^n^) of the vehicle in the local NED coordinates. State error δ**x** is propagated by the prediction step and corrected by the correction step of the EKF, and is expressed as:

(16) $$\delta x=\left[\begin{array}{c} \rho \\ \delta p^{n}\\ \delta v^{n} \end{array}\right]\;$$

where **ρ**, δ**p**^n^ and δ**v**^n^ correspond to the attitude error vector, the position error, and the velocity error, respectively.

The radar measurements, provided by the *radar reflectors matching* block described in the previous section, include the relative azimuth (*Az_j_*), elevation (*El_j_*) and range (*R_j_*) for each matched target *j*. Thus, their corresponding radar measurement equation can be written as follows:

(17) $$z_{j}=\left[\begin{array}{c} {Az}_{j}\\ {El}_{j}\\ R_{j} \end{array}\right]=\left[\begin{array}{c} {tan}^{-1}\left(\frac{p_{j}^{n}\left(2\right)-p^{n}\left(2\right)}{p_{j}^{n}\left(1\right)-p^{n}\left(1\right)}\right)\\ {sin}^{-1}\left(\frac{p_{j}^{n}\left(3\right)-p^{n}\left(3\right)}{\left|p_{j}^{n}-p^{n}\right|}\right)\\ \left|p_{j}^{n}-p^{n}\right| \end{array}\right]$$

where the known positions of the targets in local NED (*n*) coordinates are denoted as $p_{j}^{n}$, the symbol |∙∙∙| represents the vector norm, and **a**(*i*) denotes the *i*-th component of vector **a**.

The availability of **z***_j_* depends on whether target *j* has been successfully detected and matched during the implemented signal processing. Limitations such as the previously discussed insufficient azimuth resolution at long ranges, targets outside the radar FOV during the final approach phase, or weak power returns filtered during processing can reduce the number of detections. To handle this variability, the filter dynamically adjusts the dimensions of the residuals (δz), the measurement sensitivity matrix (H), and the covariance matrix (R) during the EKF correction step. Specifically, for each radar detection, the validity of the measurement is checked by verifying the effectiveness of the pairing to a specific target of interest. If valid, the corresponding residuals, measurement sensitivity matrix rows, and covariance matrix blocks are appended to their respective vectors/matrices. Therefore, the residual related to the *j*-th target is obtained by differentiating the radar measurement of Equation (17) to correct the function of the state error, yielding:

(18) $$\begin{array}{c} {\delta z}_{j}=H_{j}\delta x\\ H_{j}=\left[\begin{array}{ccc} 0_{3\times 3} & \frac{\partial z_{j}}{\partial p^{n}} & 0_{3\times 3} \end{array}\right]\\ R_{j}=\left[\begin{array}{ccc} \sigma_{Az}^{2} & 0 & 0\\ 0 & \sigma_{El}^{2} & 0\\ 0 & 0 & \sigma_{R}^{2} \end{array}\right] \end{array}$$

Here, σ*_Az_*, σ*_El_* and σ*_R_* denote the radar measurement accuracies for azimuth, elevation, and range, computed in the *radar reflector matching* block using (15). The measurement equation used in the EKF is indeed represented by Equation (18) applied to each identified target. The dimensions of the integrated residual vector (δz), measurement sensitivity matrix (*H*), and measurement covariance matrix (*R*) vary dynamically depending on the number of matched targets. For instance, one matched target results in δz of size [3 × 1], H of size [3 × 9], and R of size [3 × 3], while all the four targets detected lead the sizes to increase, respectively, to [12 × 1], [12 × 9], and [12 × 12].

The developed multi-sensor navigation architecture integrates the radar measurements into an EKF scheme, which also incorporates data from an IMU, a GNSS receiver and two cameras [47,48]. This vision-aided EKF propagates the navigation state through the IMU readings and corrects it in a loosely coupled manner through the position measurements from the camera and GNSS. Both GNSS and camera-based position information are incorporated into the filter only if they satisfy a gating condition determined by the predicted state and associated uncertainties.

**Figure 9:**
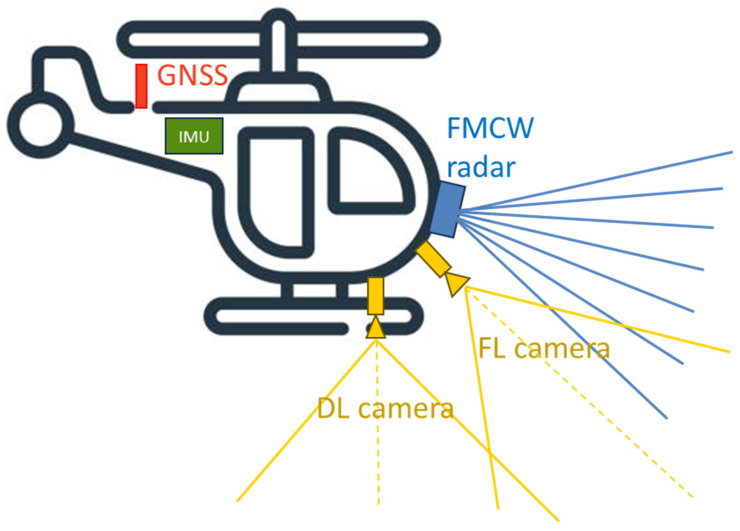
Scheme of the onboard sensors adopted in the proposed navigation architecture.

Camera data are specifically utilized during the latter portion of the flight, beginning at estimated distances of $R_{max,Cam}=200\;m$ to the landing pad. This range is determined based on the performance of the vision-aided navigation system as a function of distance. In low-visibility conditions, however, the image processing system may struggle to provide reliable measurements at $R_{max,Cam}$. In this case, the image processing tries to extract visual measurements from the acquired frames without being accepted by the gating condition until reaching the effective range, at which the navigation architecture first accepts vision-aided measurements (closer than the nominal 200 m range to the landing pad). A Convolutional Neural Network (CNN)-aided detector identifies the landing area within image frames. Once the landing area is identified, visual key points are extracted and subsequently used for pose estimation. At higher distances, these key points include features such as the vertices of the square and triangle of the landing pattern, while at closer ranges, fiducial markers are employed. These extracted key points are matched to their known NED coordinates and input into the iterative Levenberg–Marquardt (LM) algorithm. This algorithm computes the unmanned/autonomous aircraft’s relative pose to the landing site, leveraging an initial guess derived from the predicted navigation state during the EKF prediction step. To ensure continuous visual coverage of the landing pad, two cameras are installed onboard the aircraft. A FL camera configuration supports the initial vision-aided phase, while a DL configuration is optimized for the final approach phase, continuing up to touchdown (refer to Figure 9).

**Figure 10:**
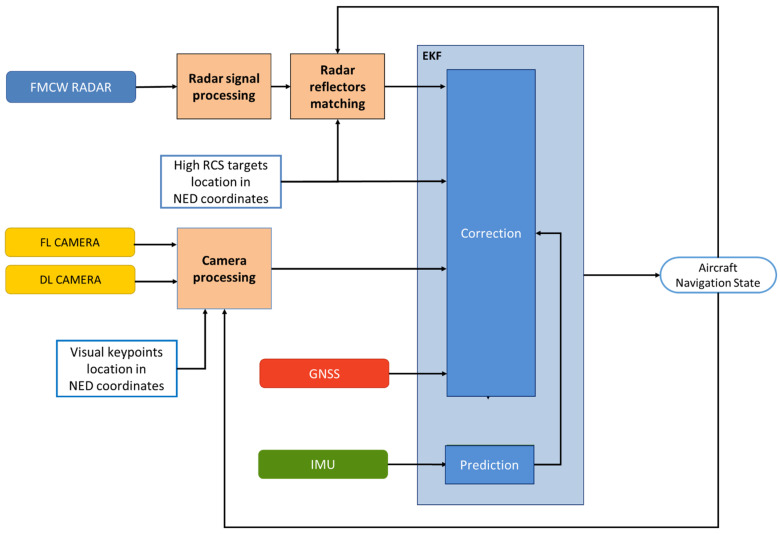
Implemented multi-sensor navigation architecture.

By tightly integrating radar data into the described EKF scheme, the architecture depicted in Figure 10 is achieved. The integration of radar within this framework provides several critical benefits. It delivers accurate position measurements at distances exceeding the operational range of vision-based sensors, ensuring reliable navigation during long-range approach phases. Furthermore, it supplements the navigation filter with an additional source of position information, enhancing system robustness under poor visibility or lighting conditions. This redundancy improves the overall integrity of the navigation system when all sensors are operational, enabling a higher degree of reliability and safety during approach and landing procedures.

The outlined navigation architecture processes data from different onboard exteroceptive sensors, enabling the definition of distinct operational modes based on the autonomous aircraft’s distance from the landing pad. These modes support the approach and landing procedures and are defined as follows:

1. Long-Range Phase. This corresponds to the time interval at which the estimated range from the landing pad is larger than $R_{max,Rad}$. In this phase, only GNSS measurements are utilized for the correction step of the EKF. Since this range exceeds the radar detection capability, it lies outside the scope of the present study and is not considered part of the final approach procedure. The lower limit of this phase is determined by the radar maximum effective detection range.
2. Radar-Aided Phase. This corresponds to the time interval at which the estimated range is between $R_{max,Rad}$ and $R_{max,Cam}$. This phase relies on radar data as a key augmentation of the GNSS-IMU fusion to ensure precise navigation during the transition towards the landing site.
3. Full multi-sensor Phase. This corresponds to the time interval at which the estimated range is smaller than $R_{max,Cam}$. At closer distances, visual measurements are also integrated into the EKF correction step. Vision-based pose estimation becomes reliable at approximately 200 m under nominal visibility conditions. Sensors’ contributions are dynamically weighted based on their estimated uncertainties, and a covariance-based gating mechanism excludes erroneous data, such as GNSS multipath errors or misidentified image features. This multi-sensor fusion ensures robust navigation, maintaining accuracy even in the event of a sensor failure. Vision-aided measurements provide the precision required for the final stages of approach, enabling the unmanned/autonomous VTOL aircraft to complete its landing procedure safely.

## 4. Simulation Environment and Results

The implemented radar signal processing pipeline and the multi-sensor navigation architecture were validated using a high-fidelity simulation environment. This section details the simulation setup and test cases, demonstrating the contribution of the radar to the overall navigation performance.

### 4.1. Simulation Environment and Test Cases

To assess the performance of the multi-sensor navigation architecture and validate the radar signal processing implementation, a comprehensive high-fidelity simulation environment was employed. Synthetic data from the autonomous VTOL aircraft sensor suite were generated as follows:

- GNSS positioning and raw IMU data are generated with the MATLAB^®^ R2024b (Natick, Apple Hill Campus, Massachusetts, United States) Navigation Toolbox;
- Camera frames are produced by linking the MATLAB^®^ R2024b UAV Toolbox to Unreal Engine 4 (UE4, Cary, 620 Crossroads Blvd, United States), which renders a customized urban environment, including the landing pattern depicted in Figure 2. The *Exponential Height Fog* actor within UE allows for the simulation of varied visibility conditions;
- For raw radar data, the high-fidelity physics-based tools Ansys (Canonsburg, Pennsylvania, United States) AVxcelerate Sensor Labs™ 2024 R1 and Ansys Asset Preparation™ 2024 R1 are used. Sensor Labs™ models the radar system, including its waveform generator, antenna arrays, analog signal conditioning, and ADC sampling. Asset Preparation™ is employed to create the radar environment, specifying the dielectric properties (and hence reflectivity) of various elements in the scenario. Together, these tools ensure the generation of realistic radar data reflecting the interaction of radar signals with the environment.

The primary parameters of the selected sensors are reported in Table 7. In the table, α represents the selected vertical deflection of the sensor in the Forward-Down plane of the aircraft reference frame. This angle indicates the sensor mounting orientation relative to a forward-facing configuration (e.g., α = 90° corresponds to a Nadir-Looking sensor). The value of the radar deflection (α = 20°) is simulated to maintain the targets of interest in the FOV in a large portion of the first phase approach trajectory (A-B in Figure 1), where higher support to vision-aided navigation is expected to be required. The selected sample rate of the IMU (i.e., 200 Hz) implies that the EKF updates the VTOL aircraft navigation state at the same frequency. The measurements of the other sensors are received at lower frequencies (i.e., 1 Hz and 10 Hz for GNSS and cameras, respectively). A complete radar acquisition consists of seven scans in elevations (each at 10 Hz) as explained within the design of the radar system, so the overall frequency of the radar system is 1.43 Hz (i.e., 10/7 Hz). Radar-aided corrections are received by the EKF only at specific elevation angles (which depend on the relative orientation between the antennas and the corner reflectors at each point of the approach trajectory), meaning that the radar measurements are received at a frequency lower than 10 Hz.

**Figure 11:**
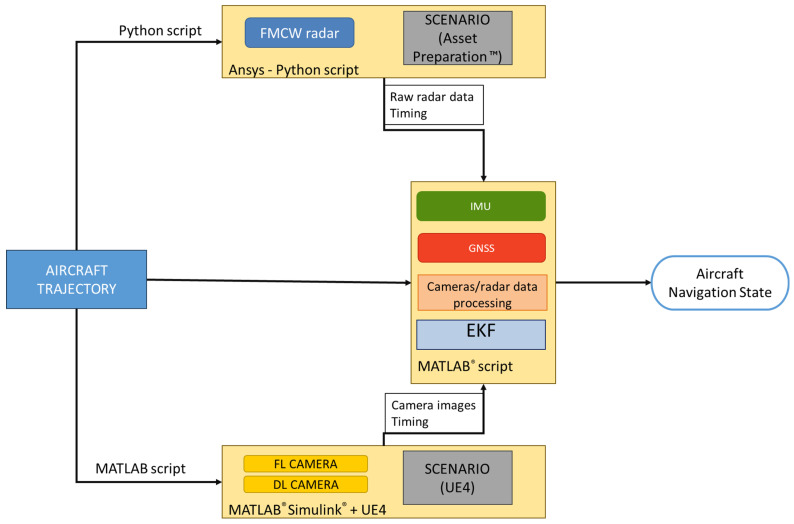
Flowchart depicting the generation of synthetic sensor data and subsequent processing.

Figure 11 reports the flowchart depicting the generation of synthetic sensor data and subsequent processing within the selected simulation environment. The unmanned/autonomous aircraft approach trajectory was sampled from the one defined in Figure 1 with a slope of ϑ=21.9°, with the motion constrained to the North-Down plane for simplicity. The data processing blocks depicted in Figure 10 (i.e., the *radar signal processing* and the consequent *radar reflector matching*, the *camera processing*, and the EKF) are implemented in a MATLAB^®^ R2024b script. The simulation environment enables the generation of variable initial estimates of autonomous VTOL aircraft navigation state, along with GNSS measurements and IMU readings, consistent with the simulated performance of these sensors. This setup allows for testing the effectiveness of the radar matching and the navigation state corrections derived from radar data detections under varying initial navigation state estimates. Realistic fog simulation aims to demonstrate the impact of FMCW radar on the multi-sensor architecture, particularly in conditions where vision-aided measurements are limited in range relative to the nominal $R_{max,Cam}=200\;m$. As shown in Table 3, the effects of water vapor on Ka-band radar are negligible for the ranges of interest. Consequently, the radar data were simulated only under nominal visibility conditions, avoiding the use of the physics-based simulator for modelling an unnecessary and complex scenario.

**Figure 12:**
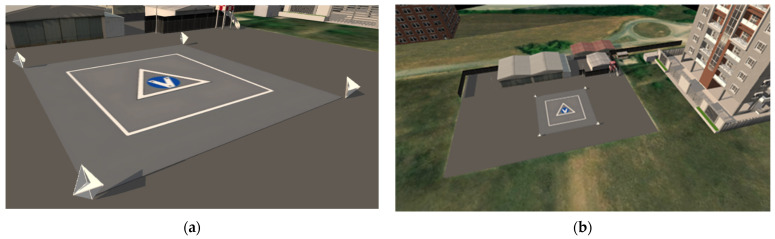
Scene generated using Ansys AVxcelerate Asset Preparation™ 2024 R1 for radar data collection: (**a**) landing pattern with four corner reflectors placed at the vertices; (**b**) scenario near the landing area, showing buildings used to simulate radar signal clutter.

The synthetic scenario generated in Ansys AVxcelerate Asset Preparation™ 2024 R1 replicates the heliport located in Aquino (Italy), a potential location for future flight tests. Four targets of interest, located at the vertices of the FATO area, are modeled as trihedral corner reflectors with the *Perfect Metal* dielectric property (Figure 12). Additionally, two nearby buildings, representing strong radar signal reflectors, are included to simulate the clutter effects typical of urban environments.

**Figure 13:**
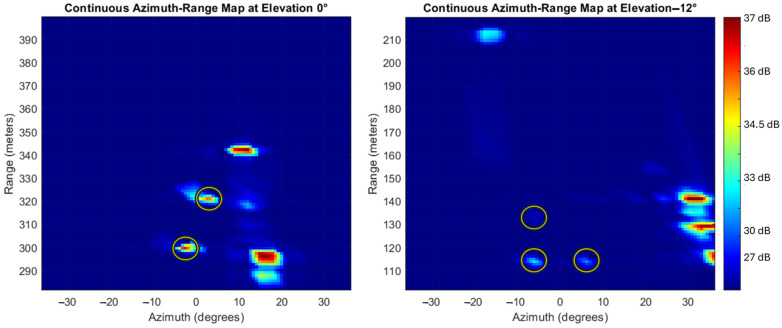
Azimuth-Range map of the received radar signal along the simulation environment. Left: slant range to the center of the landing pad: 311 m, height above the pattern: 142 m, El = 0°. Right: slant range to the center of the landing pad: 125 m, height above the pattern: 75 m, El = −12°. The intensity of the peaks is represented as a function of the SNR. Yellow rings are used to circle the targets’ reflections.

Figure 13 presents two examples of synthetic radar data, visualized as Azimuth-Range heatmaps in terms of SNR, for ranges of 311 m and 125 m at elevation angles of 0° and −12°, respectively. The target list provided by the implemented *radar signal processing* block is plotted in the heatmaps and the target reflections are highlighted using yellow rings. In the first simulated acquisition, two corner reflectors are clearly distinguishable in radar data at coordinates (Az = −4°, R = 298 m) and (Az = 4°, R = 322 m). In the second simulation, three peaks corresponding to the corner reflectors are observable, albeit with lower intensity compared to the building reflections, at coordinates (Az = −8°, R = 113 m), (Az = 8°, R = 113 m) and (Az = −8°, R = 137 m). The high SNR observed in the simulation (up to 37 dB) is due to idealized conditions, such as a simulated noise floor of −100 dBW, high transmitted power (0.1 W for 256 chirps) with coherent integration, and negligible clutter generated by the simulator in the scene. However, even in the presence of clutter, the data quality can be improved by applying Space–Time Adaptive Processing algorithms [64,65].

**Figure 14:**
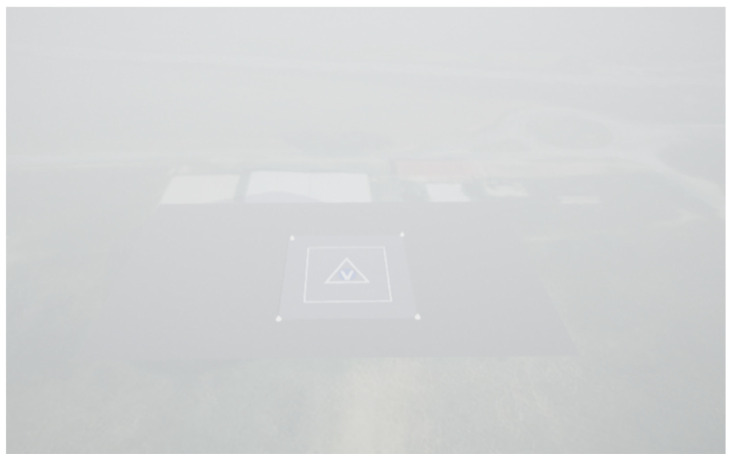
Forward-Looking (FL) camera image frame acquired through MATLAB^®^ R2021a + Unreal Engine 4.23 (UE4) under low visibility. First usable in camera processing pipeline frame under dense fog ($R_{max,Cam}=$ 89 m).

The scenario used for the generation of camera data is the same Aquino heliport area generated in UE4. As shown in Figure 11, the same trajectory selected for the simulation of radar data is applied to generate time-synchronized images, which are then processed in the MATLAB^®^ R2024b script. These images are produced under varying fog conditions, ranging from nominal visibility to dense fog that reduces the effective range for reliable measurements. Figure 14 illustrates the first frames correctly processed in the image processing pipeline under different visibility conditions.

The test cases are designed to validate the contribution of the FMCW radar to the navigation architecture and evaluate the robustness of radar and camera data processing under varying initial conditions. To this end, 100 simulations for each test case are conducted, with GNSS and IMU data randomly varied according to the parameters in Table 7 to enable a statistical analysis of the navigation filter performance. The radar contribution is highlighted by comparing the filter performance in scenarios with and without radar activation. Four test cases are defined as follows:

1. Test Case 1. Low visibility conditions (Figure 14, left) with radar activation;
2. Test Case 2. Low visibility conditions without radar activation;
3. Test Case 3. Nominal visibility conditions (Figure 14, right) with radar activation;
4. Test Case 4. Nominal visibility conditions without radar activation.

As noted, statistics on the performance navigation error metrics are evaluated over a set of 100 simulations per test case.

### 4.2. Simulation Results

**Figure 15:**
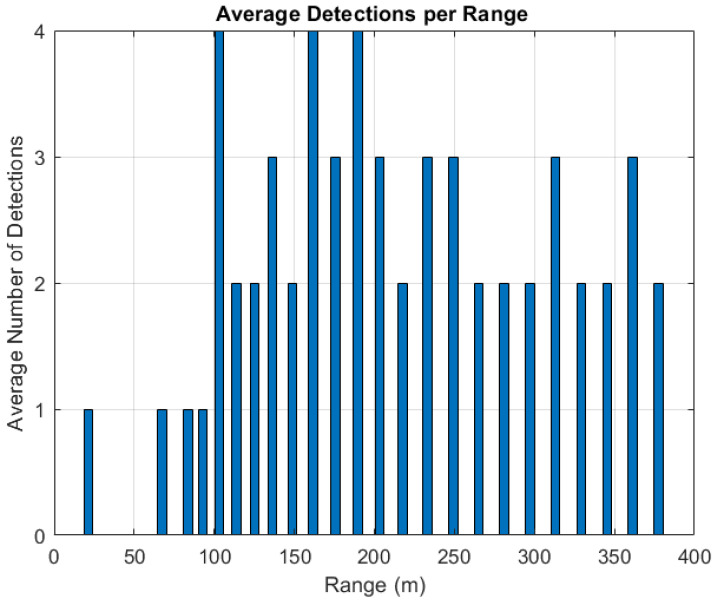
Average number of matched detections of the four corner reflectors during each complete elevation scan across all test cases.

Firstly, the average number of matched detections of the four corner reflectors along each complete elevation scan is estimated across all the test cases as a function of the range, as shown in Figure 15. These detections represent the output of the *radar reflectors matching* block of Figure 10 and demonstrate that the combination of the designed radar system and the proposed radar data processing pipeline enable at least one detected target from the beginning of the simulated approach trajectory up to about 65 m range from the landing pad. The plot reveals that the selected configuration of the corner reflectors and their relative orientation to the radar result in a limited number of returns (up to 1) in the last 100 m of the approach. This indicates that a different positioning and orientation of the corner reflectors could improve the availability of radar-aided measurements during this phase of the trajectory. Reliable detections until the touchdown could be reached by integrating further high RCS targets inside the FATO area, covered by acrylic panels to maintain the area free of any obstacle. At the same time, the mounting orientation of the radar sensor can be optimized through a gimbal to maintain the targets in view during the final phase of the approach. The distinction between the targets in the azimuth direction is achieved when at least three targets are detected during a specific acquisition, with the first two distinguished using range resolution. The plot confirms this observation, showing that during the initial phase of the trajectory (at ranges higher than 250 m), only two detections are often observed, while three detections are achieved only twice. This finding further supports the suggestion that increasing the azimuth resolution could improve the identification of corner reflectors, particularly in the early stages of the approach, as anticipated when specifying the required angular resolution performance. Finally, the absence of false detections along each simulated case is highlighted to confirm the robustness of the pipeline in correctly matching the detected targets.

While the previous analysis focuses on radar signal processing, the following ones evaluate the performance of the complete multi-sensor navigation architecture. Specifically, various plots of the positioning errors of the EKF are presented as a function of the range to the landing pad. Firstly, the results of the statistical analysis are presented and the radar contribution to the architecture is highlighted. The positioning errors along the approach trajectory are compared to the Required Navigation Performance (RNP) during the AAM approach trajectory, preliminarily evaluated using the technique described in [66]. In this work, the required Horizontal and Vertical Protection Level (HPL/VPL) of the landing unmanned/autonomous aircraft are defined at a point corresponding to the equivalent of point B in Figure 1 (i.e., where the descent slope steepens for the final phase of the procedure). This accuracy requirement is linearly interpolated to the corresponding requirement in cruise [67] (i.e., at the point A placed at the beginning of the simulated approach procedure, HPL = 8 m and VPL = 13 m). The specific RNP is evaluated through the Horizontal and Vertical Position Error (HPE/VPE), representing the actual navigation system errors along the horizontal and vertical directions. The HPE corresponds to the Euclidean distance combining the North (*N*) and East (*E*) errors, while the VPE is the error along the Down (*D*) axis. The HPE and VPE correspond to the 95% bounds of the horizontal (*H*) and vertical (*V*) positioning errors and are, therefore, estimated along the approach trajectory through the statistical analysis for each test case as the mean of the error (*μ*) plus twice its standard deviation (*σ*):

(19) $$\begin{array}{c} HPE=\left|\mu_{H}+2*\;\sigma_{H}\right|\leq HPL\\ VPE=\left|\mu_{V}+2*\;\sigma_{V}\right|\leq VPL\\ where\;H\equiv \;\sqrt{N^{2}+E^{2}},\;\;\;V\equiv D \end{array}$$

**Figure 16:**
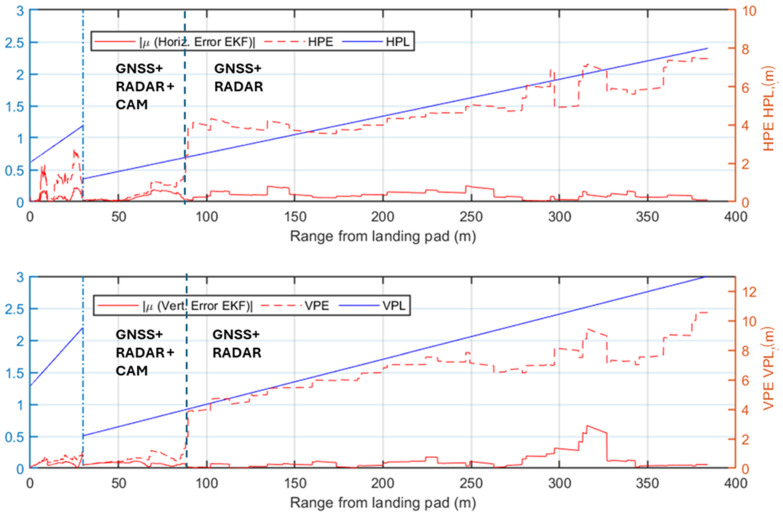
Test Case 1: mean of the EKF error and Horizontal/Vertical Position Error (H/VPE) bounds compared with Horizontal/Vertical Protection Level (H/VPL) as a function of the range from the landing pad. Results obtained from a statistical analysis. The plot scale is enlarged in the last 30 m to highlight the errors in the final phase of the approach.

The first selected test case considers low visibility conditions and the radar system actively providing measurements to the EKF. The HPE and VPE results, shown in Figure 16, confirm the radar contribution to maintaining navigation accuracy, as the errors generally of the same order of magnitude or well below the supposed HPL and VPL. Specifically, in the phase where the camera is not available, the radar measurements significantly reduce positioning errors below the limits. However, between ranges of approximately 85 m and 150 m, the horizontal error slightly exceeds the HPL, highlighting that the defined angular and range resolution are minimum requirements for a multi-sensor architecture. Eventually, the error in the navigation solution decreases further when the camera begins to process image frames, providing highly accurate vision-aided position measurements. In this phase, both HPE and VPE remain well below the defined HPL and VPL.

**Figure 17:**
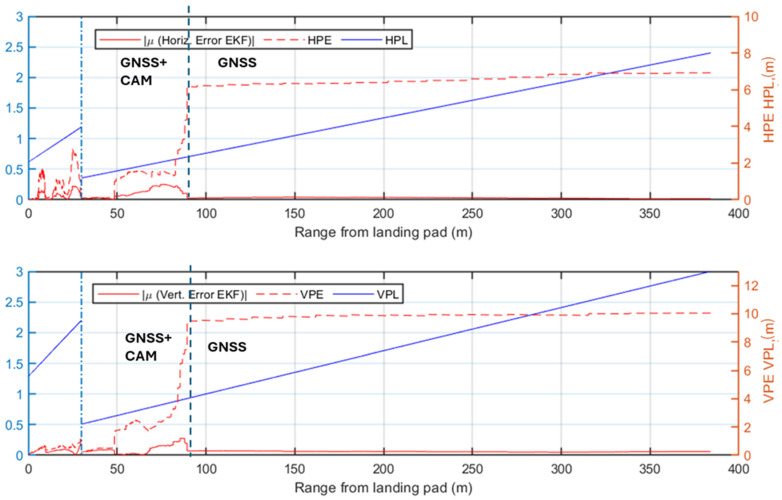
Test Case 2: mean of the EKF error and H/VPE bounds compared with H/VPL as a function of the range from the landing pad. Results obtained from a statistical analysis. The plot scale is enlarged in the last 30 m to highlight the errors in the final phase of the approach.

Figure 17 presents the positioning errors for Test Case 2, which shares the same visibility conditions as Test Case 1 but excludes radar detections from the navigation architecture. The HPE/VPE indicate that GNSS-based corrections alone are insufficient to meet the stringent accuracy requirements, thereby justifying the integration of radar into the architecture. Although the final vision-aided approach phase (last 85 m) meets the HPL/VPL, the earlier phase largely exceeds the defined limits, demonstrating the risks of conducting approach procedures under similar conditions without radar support.

**Figure 18:**
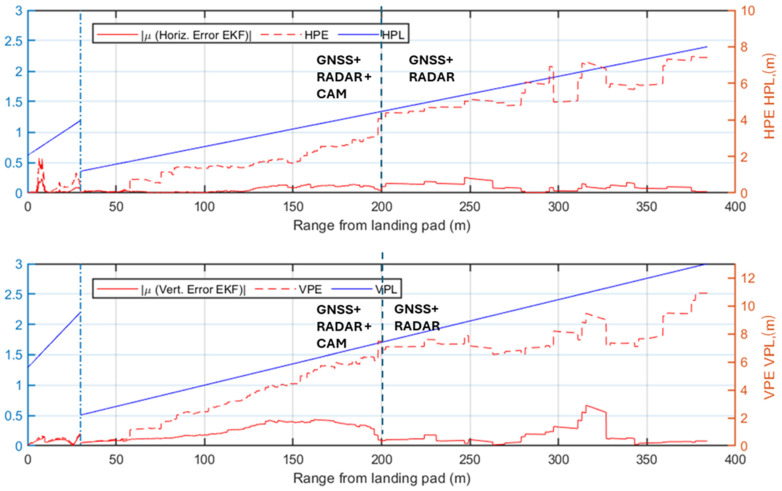
Test Case 3: mean of the EKF error and H/VPE bounds compared with H/VPL as a function of the range from the landing pad. Results obtained from a statistical analysis. The plot scale is enlarged in the last 30 m to highlight the errors in the final phase of the approach.

The first two test cases demonstrate the radar’s significant benefit to navigation system performance in low visibility conditions. An analysis of the nominal visibility conditions scenario is also reported to validate the proposed architecture in these cases. The first test case under nominal visibility (i.e., Test Case 3), shown in Figure 18, integrates radar into the processing pipeline. The HPE and VPE indicate that the implemented system respects the imposed accuracy bounds, up to two points in the horizontal direction at ranges of 300 m and 320 m. However, the resulting HPE for both points overcomes the supposed HPL only for a few centimeters and a reduced amount of time. These two points, also observed in Test Case 1, result from the slightly inaccurate matching of one of the corner reflectors, which is paired to an adjacent cell with a higher return (3 m away from the actual cell containing the correct target). Nevertheless, the overall performance of the system validates the accuracy of the implemented multi-sensor architecture.

**Figure 19:**
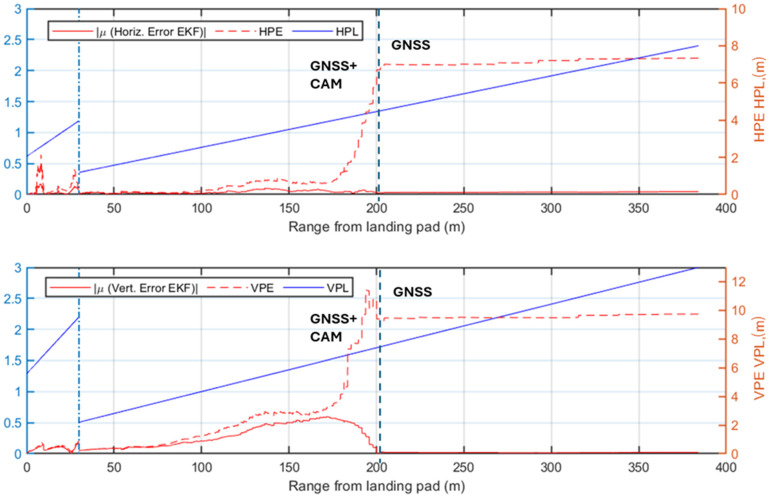
Test Case 4: mean of the EKF error and H/VPE bounds compared with H/VPL as a function of the range from the landing pad. Results obtained from a statistical analysis. The plot scale is enlarged in the last 30 m to highlight the errors in the final phase of the approach.

Figure 19 illustrates the HPE and VPE in Test Case 4, where only GNSS and visual measurements are integrated into the navigation filter, and the camera provides reliable position estimates from a range of 200 m from the landing pad. The system errors show that the performance of the selected GNSS receiver faces challenges in the initial phase of the approach procedure until the camera improves the navigation solution. The comparison of these results with the radar-aided case in Figure 18 highlights the radar contribution in the nominal visibility case, where it ensures compliance with the assumed navigation requirements.

**Figure 20:**
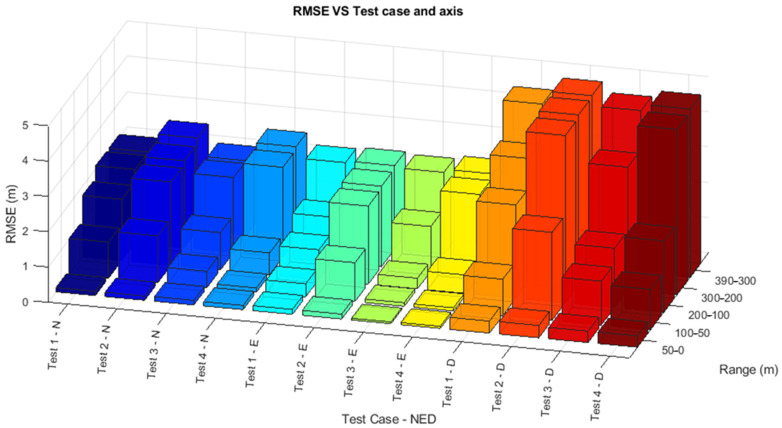
A 3D histogram of the RMSE along the 3 axes in function of the range across the 4 test cases.

The statistics of the EKF navigation error across the four test cases are summarized in Table 8 by using the Root Mean Square Error (RMSE) computed along the NED directions. The RMSE values corroborate the earlier observations demonstrating improved navigation performance when radar data are actively contributing to the system. The differences in errors between the across-track direction (East) and the along-track and vertical directions (North and Down) are attributed to the relatively low elevation accuracy performance of the radar. Notably, the centimeter-level RMSE values in the final 50 m emphasize the critical role of camera data in maintaining high navigation accuracy across all test cases. The visualization of the data in the form of a 3D histogram in the function of the range is shown in Figure 20.

For a more detailed analysis, the positioning errors along the North, East, and Down axes are examined in single-shot simulations. These simulations use a fixed random seed in MATLAB^®^ R2024b to ensure consistent GNSS measurements and IMU readings across all four test cases. In this way, variations arise only from the presence or absence of radar measurements and the camera activation range, depending on visibility conditions.

**Figure 21:**
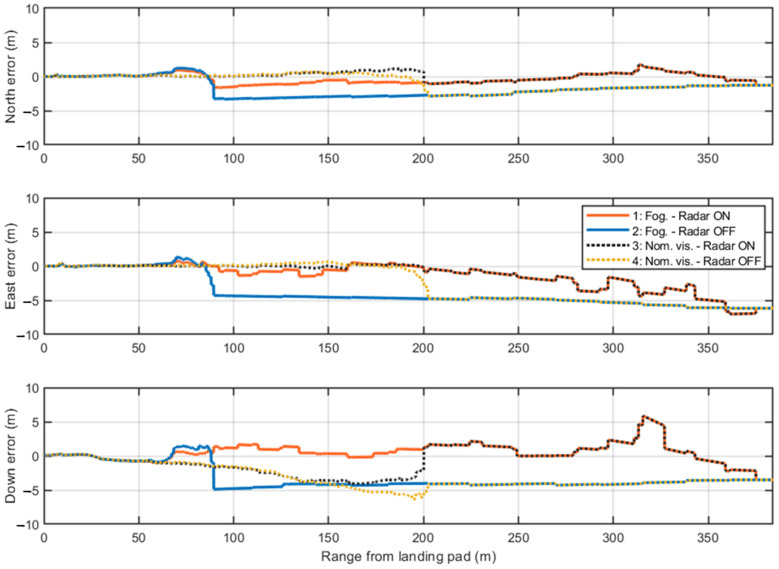
Single-shot Case 1. EKF-based Positioning Error along the N–E–D axes for all the 4 test cases.

Figure 21 presents the first single-shot simulation. The initial GNSS measurements show consistent errors along the East and Down axes, which are mitigated when radar data provides corrections to the navigation filter. The only exception occurs along the Down component at approximately 320 m, where the previously noted issue (in Figure 17) arises due to the incorrect association of one corner reflector to the corresponding range cell. This error causes the filter correction step to estimate a slightly higher vertical position than the actual one. Nevertheless, the radar enhances navigation accuracy up to the range where the camera becomes active in both visibility scenarios. The overlap between Test Cases 1–3 and 2–4 before the camera activation is due to the identical seed used in the single-shot simulations. These pairs of cases differ only in the range at which the camera starts providing visual measurements and in the processed image frames. The positioning errors in the final 60 m confirm the high accuracy of the visual measurements, coupled with low covariance, which leads the EKF to primarily trust the camera processing during this phase.

**Figure 22:**
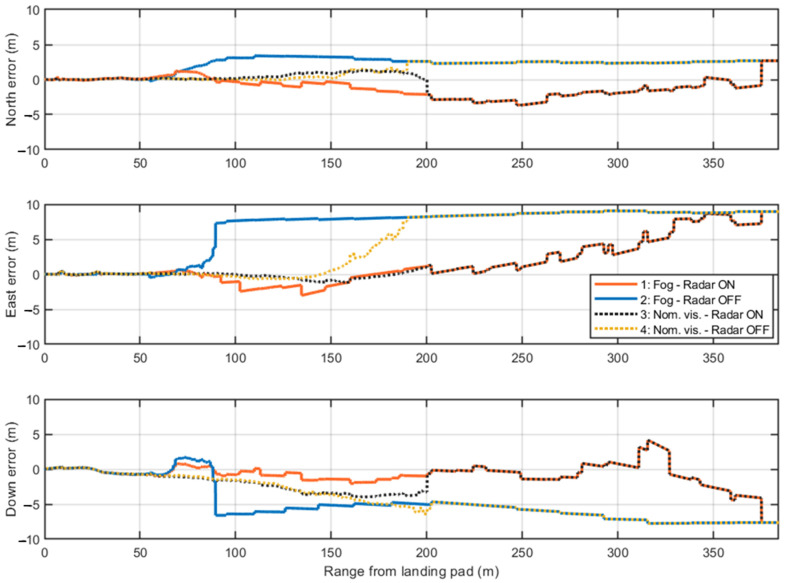
Single-shot Case 2. EKF-based Positioning Error along the N–E–D axes for all 4 test cases.

The integration of radar measurements into the EKF also corrects coarser errors in GNSS measurements. This correction is evident in Figure 22, where the starting GNSS error along the N–E–D components is about 3.5 m, 9 m, −8 m, respectively. These errors persist in the non-radar cases until camera measurements are integrated. Conversely, in radar-aided cases, the EKF benefits from corrections starting with the first radar acquisition, resulting in acceptable positioning errors across all three components. The gradual improvement along the East axis is linked to the radar limited azimuth resolution, as previously discussed in relation to Figure 15. This limitation initially delays clear separation of corner reflectors within azimuth cells, which postpones corrections along the cross-track direction.

**Figure 23:**
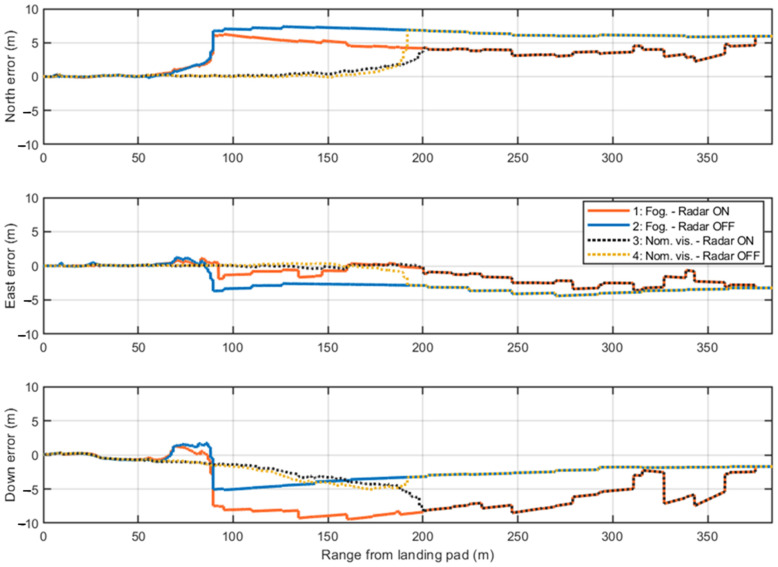
Single-shot Case 3. EKF-based Positioning Error along the NED axes for all 4 test cases.

The final presented single-shot simulation, shown in Figure 23, highlights a shortcoming of the designed radar system that impacts the North and Down axes positioning estimates of the EKF. Specifically, when the initial errors are positive along the North direction and negative along the Down, the radar-aided corrections face challenges in addressing these errors as effectively as in the previously presented cases. This shortcoming arises from the radar elevation accuracy, which does not provide enough information to resolve the limited capacity to distinguish North and Down errors. Consequently, the radar cannot correctly estimate that the unmanned/autonomous aircraft is closer along the North direction and higher above the landing pad than the initial position suggested by the incorrect GNSS measurements. Despite this misalignment, the radar still estimates the same range from the landing pad, and consequently, the EKF encounters challenges in efficiently correcting the errors. Two potential strategies can be employed to address this poor observability of North and Down errors. The first strategy is improving the elevation resolution of the radar system, while the second involves integrating an altimeter (e.g., laser one) combined with a 3D model of the urban area into the multi-sensor architecture. The altimeter would provide accurate height estimates, enabling corrections along the Down axis. Since the range to the corner reflectors is derived from FMCW radar measurements, correcting the Down axis would also improve the accuracy of the North axis, ensuring consistency with the estimated range.

## 5. Conclusions and Future Works

This study demonstrates the feasibility and additional value of integrating a radar system in multi-sensor navigation architectures optimized for unmanned/autonomous AAM approach and landing operations. In the proposed architecture, the radar provides significant benefits by enhancing navigation accuracy up to the activation of the camera, a feature particularly valuable in low-visibility conditions and at high ranges from the landing area. The obtained precise navigation is essential to ensure safety and efficiency during autonomous aircraft or drone operations, especially in complex urban environments. The key contributions of the work are related to the definition of the requirements of the radar system and its contribution to the proposed multi-sensor architecture.

Firstly, the minimum radar system requirements (i.e., range resolution of 11.26 m, azimuth resolution of 4.6° and elevation beamwidth of 15.1°) are derived from realistic trajectory and infrastructure constraints, ensuring their practical applicability to AAM scenarios. The selected ground infrastructure adheres to VFR operation guidelines and is adaptable for manned and unmanned approach procedures. The SWaP characteristics of the radar system emerging from these requirements demonstrate the feasibility of installing such systems on AAM platforms. Specifically, a preliminary radar system is designed working with the Ka-band, implying that the required radar antenna size results of about 40 cm^2^ and the minimum transmitted power is less than 1 W.

Subsequently, a complete radar signal processing pipeline is developed, transforming raw radar data into usable inputs for the navigation filter in a tightly coupled manner. The pipeline proves robust in identifying the high-RCS targets within the vertiport area in the range of distances between 400 m and 100 m from the landing area, even within cluttered environments. The tight integration of the matched detections in the EKF also enables the extraction of navigation information from a limited number of recognized targets. The selection of a high-fidelity simulation environment enables the testing of the proposed navigation architecture with realistic synthetic sensor data, including the designed FMCW radar. The simulation results show that the integration of radar measurements significantly improves navigation performance, particularly in low-visibility conditions, enabling the system to meet stringent accuracy requirements that GNSS corrections alone could not achieve.

Although the designed radar system proves effective, its resolution in azimuth and elevation could be enhanced to refine position estimates and address potential ambiguities. Improving the radar resolution would allow the navigation system to meet stringent accuracy requirements closer to the landing pad, similar to the progression between categories in ILS approaches. At its current state of design, the radar system’s equivalent category does not yet enable reaching the touchdown point without visual contact (provided by cameras) with the landing pad.

Future work will focus on the design and optimization of high-RCS targets to further improve radar performance. The preliminary radar design already integrates the autonomous detection of potential intruders flying in the proximity of the vehicle, enabling Sense-and-Avoid (SAA) operations during approach and landing. Incorporating this functionality into the multi-sensor architecture could provide warnings in case of potential collisions, further enhancing the system’s safety capabilities.

In conclusion, this study highlights the feasibility and potential of radar in supporting robust navigation solutions under challenging conditions, paving the way for reliable and weather-resilient highly autonomous AAM operations in landing scenarios.

## Figures and Tables

**Table 1:** Airborne radar frequency bands characteristics.

Band	Frequency Range [GHz]	Center Frequency *f*_c_ [GHz]	Wavelength *λ* [cm]
Ku	13.25–13.40	13.325	2.25
K	24.45–24.65	24.55	1.23
Ka	32.30–33.40	32.85	0.91
W	92.00–95.50	93.75	0.32

**Table 2:** Free-space path loss in different radar frequency bands.

Band	*f*_c_ [GHz]		$\gamma_{FSPL}\left[dB\right]$	
		100 [m]	200 [m]	400 [m]
Ku	13.325	94.94	100.96	106.98
K	24.45	100.25	106.27	112.29
Ka	32.85	102.78	108.80	114.82
W	93.75	111.89	117.91	123.93

**Table 3:** Atmospheric loss coefficient in different radar frequency bands.

Band	*f*_c_ [GHz]	*γ_atm_* [dB/km]
Ku	13.325	0.0218
K	24.55	0.1606
Ka	32.85	0.0955
W	93.75	0.4134

**Table 4:** Rain loss coefficient in different radar frequency bands and selected rain fall rates.

Band	*f*_c_ [GHz]		$\gamma_{r}\left[dB/km\right]$	
		2 [mm/h]	4 [mm/h]	6 [mm/h]
Ku	13.325	0.1632	0.3314	0.5024
K	24.45	0.6119	0.1377	1.6367
Ka	32.85	1.0788	1.9096	2.6686
W	93.75	3.4630	5.3174	6.8356

**Table 5:** Antenna size for different radar frequency bands—$N_{x}=24,$ $N_{y}=8,$ d=λ/2.

Band	Center Frequency *f*_c_ [Ghz]	Wavelength *λ* [cm]	Antenna Size [cm^2^]
Ku	13.325	2.25	9 × 27
K	24.55	1.23	4.9 × 14
Ka	32.85	0.91	3.7 × 11
W	93.75	0.32	1.3 × 3.9

**Table 6:** Minimum required transmitted power for different radar frequency bands for *R* = 400 m.

Band	*λ* [cm]	$P_{TX}^{min}\left[W\right]$
Ku	2.25	0.3760
K	1.23	0.6928
Ka	0.91	0.9270
W	0.32	2.6456

**Table 7:** Key parameters of the simulated sensors.

Sensor	Parameter	Value
IMU	Accelerometer Velocity Random Walk (VRW)	0.6 m/s/sqrt (h)
	Accelerometer Bias Instability (ABI)	0.50 mg
	Gyroscope Angular Random Walk (ARW)	0.05 deg/sqrt (h)
	Gyroscope Bias Instability (BI)	0.6 deg/h
	Sample Frequency	200 Hz
GNSS receiver	GNSS Position Standard Deviation	2.5 m Horizontal 5 m Vertical
	Sample Frequency	1 Hz
FMCW radar	Center frequency	Ka-band, 32.85 GHz
	Tx Array	1 × 8 antennas
	Rx Array	24 × 8 antennas
	Range resolution, ΔR	3 m
	$\mathrm{Angular}\;\mathrm{resolutions}\;(\mathrm{Az},\;\mathrm{El}),\;\Theta_{Az}\;and\;\Theta_{El}$	4°, 12°
	Mounting Angle α	20°
	Elevation Scan Rate	10 Hz
	Image Size [pixels]	[1920, 1200]
	Principal Point [pixels]	[960, 600]
FL camera	Focal Length [pixels]	[1365, 1365]
	Mounting Angle *α*	45°
	FOV (Az, El)	[81°, 50°]
	Frame Rate	10 Hz
	Image Size [pixels]	[2048, 2048]
	Principal Point [pixels]	[1024, 1024]
DL camera	Focal Length [pixels]	[1181, 1181]
	Mounting Angle *α*	90°
	FOV (Az, El)	[99°, 99°]
	Frame Rate	10 Hz

**Table 8:** Root Mean Square Error (RMSE) on the North–East–Down (NED) axes computed at different range intervals on the statistical analysis. The RMSE is evaluated along the 4 considered test cases through 100 simulations per test case.

Test Case				EKF—RMSE [m]		
		390 m < *R* < 300 m	200 m < *R* < 300 m	100 m < *R* < 200 m	50 m < *R* < 100 m	0 m < *R* < 50 m
**1.**	N	2.09	2.09	1.74	1.02	0.12
Fog,	E	2.32	1.27	0.84	0.37	0.14
Radar ON	D	4.51	3.46	2.64	1.03	0.34
**2.**	N	2.62	2.47	2.36	1.33	0.12
Fog,	E	2.37	2.28	2.21	1.10	0.14
Radar OFF	D	4.88	4.83	4.74	2.49	0.35
**3.**	N	2.11	2.13	1.06	0.46	0.13
Nominal visibility,	E	2.31	1.27	0.30	0.10	0.05
Radar ON	D	4.59	3.48	1.68	1.24	0.32
**4.**	N	2.64	2.55	0.62	0.12	0.12
Nominal visibility,	E	2.40	2.31	0.52	0.09	0.06
Radar OFF	D	4.77	4.68	2.02	1.14	0.32

## Data Availability

The datasets presented in this article are not readily available because the data are part of an ongoing study. Requests to access the datasets should be directed at Paolo Veneruso.
